# The Regionalization of National-Scale SPARROW Models for Stream Nutrients^1^

**DOI:** 10.1111/j.1752-1688.2011.00581.x

**Published:** 2011-10

**Authors:** Gregory E Schwarz, Richard B Alexander, Richard A Smith, Stephen D Preston

**Keywords:** stochastic models, nutrients, watershed management, statistics, geospatial analysis, transport and fate, nonpoint source pollution, computational methods

## Abstract

**Abstract:**

This analysis modifies the parsimonious specification of recently published total nitrogen (TN) and total phosphorus (TP) national-scale SPAtially Referenced Regressions On Watershed attributes models to allow each model coefficient to vary geographically among three major river basins of the conterminous United States. Regionalization of the national models reduces the standard errors in the prediction of TN and TP loads, expressed as a percentage of the predicted load, by about 6 and 7%. We develop and apply a method for combining national-scale and regional-scale information to estimate a hybrid model that imposes cross-region constraints that limit regional variation in model coefficients, effectively reducing the number of free model parameters as compared to a collection of independent regional models. The hybrid TN and TP regional models have improved model fit relative to the respective national models, reducing the standard error in the prediction of loads, expressed as a percentage of load, by about 5 and 4%. Only 19% of the TN hybrid model coefficients and just 2% of the TP hybrid model coefficients show evidence of substantial regional specificity (more than ±100% deviation from the national model estimate). The hybrid models have much greater precision in the estimated coefficients than do the unconstrained regional models, demonstrating the efficacy of pooling information across regions to improve regional models.

## Introduction

Water managers increasingly have a need for model-based information to address eutrophication problems in large watersheds and their coastal receiving waters (e.g., Chesapeake Bay, Connecticut River Basin and Long Island Sound, Mississippi River Basin and Gulf of Mexico) where the development of effective solutions is complicated by the diversity of nutrient contributions from upstream sources and catchments (New York State Department of Environmental Conservation and Connecticut Department of Environmental Protection, 2000; Koroncai *et al.*, 2003; Mississippi River/Gulf of Mexico Watershed Nutrient Task Force, 2008). Managing the watershed response at these large scales places multiple demands on the water-quality model. The model must provide an accurate assessment of water-quality conditions for locations that are deficient in monitoring data; the model must make an accurate determination of the relative importance of an extensive list of nutrient sources contributing to a particular water-quality impairment, many of these sources being physically distant from the impaired area; and the model must adequately describe how the aquatic system will respond to hypothetical changes in nutrient sources or factors affecting their transport.

The U.S. Geological Survey's (USGS) SPAtially Referenced Regressions On Watershed attributes (SPARROW) model – a hybrid statistical and process-based mass balance model – was developed to explain spatial variability in mean water-quality conditions and predict source contributions in the streams of large, heterogeneous basins (Schwarz *et al.*, 2006; Preston *et al.*, 2009) (see also the brief description of the SPARROW methodology contained in the Supporting Information for this article). SPARROW has been used previously to assess nutrient loadings in large watersheds in the United States (U.S.) (e.g., Smith *et al.*, 1997; Alexander *et al.*, 2008) and other countries (Alexander *et al.*, 2002). An important question in applying SPARROW and other water-quality models in large watersheds is how the governing material transport equations should differ spatially in their functional forms and/or coefficients to account for the effects of landscape heterogeneity and spatial scaling on nutrient supply and transport processes. Despite advances in watershed science to address this question, uncertainties remain over the nature of these effects and how they should be represented in watershed models (e.g., McDonnell *et al.*, 2007; Kirchner, 2009). Existing national-scale SPARROW nutrient models are estimated with the assumption that the coefficients of the model that describe the marginal effect of water-quality explanatory variables on stream nutrient loads (e.g., the loading response from an incremental change in soil permeability; the change in the fraction of nutrient removed in streams from a unit change in water travel time) are the same for all locations nationally (Alexander *et al.*, 2008). This assumption insures that the widest ranges of environmental conditions are used to estimate the effects of individual model processes, and imparts a parsimonious form that facilitates the interpretation of the estimated coefficients. The assumption may be overly simplistic, however, if there exist latent processes, related to landscape heterogeneity or process interactions, for which surrogate measures of their effects are not available in geospatial datasets. Under these conditions, large-scale model development could lead to biased predictions in particular regions and under certain management or forecasting scenarios.

In recognition of potential regional variation in water-quality model processes, the USGS National Water Quality Assessment (NAWQA) Program has developed a set of SPARROW regional major river basin (MRB) nutrient models, described in this Featured Collection (for a summary of these models, see Preston *et al.*, this issue). The estimation of these regional models has benefited from a large assemblage of water-quality data, representing an approximate order of magnitude increase in the number of monitoring sites, across all regions, compared to the number of sites from which data were available to construct existing national-scale SPARROW nutrient models (Smith *et al.*, 1997; Alexander *et al.*, 2008). The inherent flexibility afforded by independent regional models allows the predictions of current water-quality conditions derived from these models to have less bias and greater precision than those of national models; thus, from the standpoint of water-quality assessment, the regional models out-perform the national models.

Regionalization of models, however, also poses a potential negative consequence: an individual regional model likely encompasses a narrower range in basin attributes than does a national model. For example, a climate variable typically exhibits less variation within a region than across the entire nation. In a model, an attribute's effect on water quality is mediated by a coefficient, the estimation of which is made more precise the greater the variation of the attribute in the sample of data used to estimate the model. The reduced variation of an attribute within a confined region thereby reduces the precision of the attendant coefficient. Thus, users of a regional model (e.g., water resource managers) face a fundamental tradeoff in model accuracy between the bias and precision of the model predictions. On the one hand, if a model coefficient's true value varies across regions, the loss in precision due to less within-region variability in the associated basin attribute may be an acceptable outcome for obtaining an unbiased coefficient estimate. On the other hand, if the true coefficient does not vary across regions, the reduced precision implies a loss in accuracy of the estimated coefficient, a loss that possibly could be ameliorated by expanding the spatial extent of the model to include a wider range of basin attributes. It remains an open question, therefore, whether the advantage of added flexibility afforded by individual regional models, which facilitates a less biased assessment of existing water-quality conditions, outweighs the loss of precision, which potentially diminishes a water manager's ability to use the model for predicting changes in water quality brought about by changes in its causative factors.

This article has two major objectives: the first objective is to evaluate regional variation in the nutrient model coefficients using a “fixed-effects” approach (Judge *et al.*, 1985) to determine whether a single national model should be rejected in favor of a more regional-based approach. The “fixed-effects” approach to regionalization requires specifying multiple sets of coefficients in the national model, a separate set for each region of the analysis, with each regional set consisting of all the process coefficients included in the national model. This approach is a generalization of the fixed effects approach commonly used in longitudinal studies, which typically limit the fixed effects specification to an intercept term. Functionally, with fixed-effects specified for all process coefficients, the approach is equivalent to estimating independent regional models, the manner of regionalization implemented by the SPARROW MRB models. The fixed-effects method has been used, to varying degrees, in numerous previous SPARROW modeling studies (for examples of partial approaches, whereby fixed effects are specified only for a subset of the process coefficients, see Smith *et al.*, 2003; Hoos and McMahon, 2009). An advantage of the approach is its flexibility – it places no restrictions on the pattern of variation taken by the regional process coefficients. The chief disadvantage, as mentioned above, is that the precision of the coefficient estimates of the independently estimated models may be compromised if there is too little within-region variation in basin attributes.

The second major objective of this article is to evaluate the potential for improving the precision of the regional coefficient estimates. As suggested above, if it is known that regional coefficients are similar, it may be possible to improve precision by pooling information across regions. In the context of the fixed-effects approach, one way to do this is to estimate the model with cross-region constraints placed on the coefficients. The resulting model, which we call a hybrid model, effectively pools all the region-specific information, allowing the estimation of process coefficients that are not statistically different across regions to benefit from the greater variation in basin attributes afforded by a national-scale analysis. The constrained estimates of the fixed-effects coefficients retain some degree of regional variation, with the extent of variation depending on the particular cross-region constraints imposed as part of the specification of the hybrid model.

A distinct advantage of the hybrid model, from the standpoint of the analysis described here, is that it encompasses both the regional fixed-effects model and the national model. At one extreme, if there are no constraints, the hybrid model is identical to the regional fixed-effects model, the model that is functionally equivalent to specifying independent regional models. At the other extreme, if all possible independent constraints are applied, the hybrid model becomes identical to the national model. Thus, by partially constraining the regional fixed-effects model, the hybrid model provides a framework for evaluating the tradeoff between achieving prediction accuracy and improving coefficient precision in a regional context.

**FIGURE 1:**
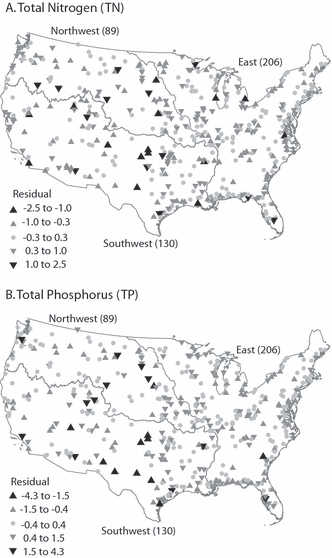
The Residuals Estimated From the 1992 National SPARROW Models for (A) Total Nitrogen and (B) Total Phosphorus (Alexander *et al.*, 2008). The residuals for total nitrogen are weighted to account for uncertainty in the monitored load estimates (Alexander *et al.*, 2008, p. S-12). A negative residual implies overprediction of the SPARROW model. The number of monitoring sites in the region is given in parentheses.

The national models serving as the subject of the fixed-effects analysis are the previously published SPARROW models for total nitrogen (TN) and total phosphorus (TP), estimated for the base year 1992 (Alexander *et al.*, 2008). As our interest is to isolate the effects of regionalization, yet retain as much as possible the specifications of the existing national models, this analysis uses data for the same set of monitoring stations that were used by Alexander *et al.* (2008). This collection of stations is too small, however, to obtain reasonable regional estimates for the number of regions used in the NAWQA MRB regional studies, which are based on many more stations. To compensate, this study adopts a rather coarse partitioning of the nation into only three regions: the East, Northwest, and Southwest regions of the conterminous U.S. (see Figure 1). This coarse regionalization makes it unlikely that the full range of regional effects present in the U.S. are properly accounted for, and supporting evidence of this shortcoming is presented. The limited size of the dataset used in this analysis also makes it likely that some of the regional variation exhibited by the hybrid model coefficient estimates is spurious. For these reasons, it is prudent to view the hybrid model coefficient estimates presented here as preliminary. The subsequent production of “management-ready” coefficient estimates should be possible by applying the hybrid method to the expansive set of data incorporated in the NAWQA MRB studies, the analysis of which will likely also benefit from altering the specification of the models beyond the existing national SPARROW models. Despite the shortcomings, we believe the data are sufficient to demonstrate the potential utility of moving beyond modeling frameworks that are either exclusively national or strictly regionally independent.

The following analysis begins with a brief description of the recently published national nutrient SPARROW models (Alexander *et al.*, 2008), developed for the 1:500,000-scale Reach File 1 network (Nolan *et al.*, 2002). An initial evaluation of the national nutrient models is undertaken to determine if regional fixed effects are present in three major regions of the conterminous U.S., with attention given to whether the regional effects can be associated with specific groups of transport processes that are an intrinsic feature of a SPARROW model specification. A subsequent section describes a practical approach for identifying constraints to be applied to the regional fixed-effects model to estimate the hybrid model. A section describing the results of the estimation of the hybrid model follows, with emphasis placed on demonstrating the effects of agglomerating information across regions on the precision of coefficient estimates and model predictions. The concluding sections contain a discussion of regionalization methodology in the context of other approaches found in the literature and a summary of the findings of this study. All mathematical derivations used in the analysis are described in an Appendix and in the Supporting Information for this article. Additionally, the Supporting Information includes a brief description of the SPARROW model and a detailed presentation of results from the regional fixed-effects and hybrid models.

## The National SPARROW Nutrient Models

The specific TN and TP SPARROW models serving as the subject of the regionalization analysis are described in Alexander *et al.* (2008). Both models adopt 1992 as the base year for the analysis, and use the spatial infrastructure provided by the recent version of the enhanced Reach File 1 (E2RF1) stream network (Nolan *et al.*, 2002), a 1:500,000-scale network consisting of approximately 65,000 reach segments in the conterminous U.S., including digital elevation-delineated catchments for each reach.

The SPARROW modeling approach groups the determinants of water quality into three types: source variables that identify specific sources of contaminant, land-to-water variables that mediate the amount of contaminant source that is transported to streams included in the digital stream network, and aquatic removal rate variables that determine the amount of contaminant that is transported through streams in the digital network (a detailed description of the SPARROW methodology is contained in the Supporting Information; see Schwarz *et al.*, 2006). The nutrient sources used in the national nutrient models include long-term mean atmospheric deposition, detrended to 1992 (TN model only); 1990 population (an urban source surrogate); 1992 National Land Cover Data estimates of forested land, barren land, and shrub land; and 1992 manure and fertilizer nitrogen and phosphorus loading to agricultural lands separately identified as growing corn and soybeans, alfalfa, wheat (TN model only), and other crops. The land-to-water delivery variables include soil permeability, drainage density (TN model only), mean temperature (TN model only), mean precipitation, specific catchment area (the catchment average of the area upslope of each grid cell within the catchment, divided by the average cell width; see the Supporting Information of Alexander *et al.*, 2008), percent of area artificially drained, and mean catchment slope (TP model only). The specified models include proportionate loss processes in reach network stream and reservoir segments. Both the stream and reservoir processes are based on empirically estimated mean settling velocities, a conceptual mechanism affecting the mean rate at which contaminants are removed from the aquatic environment (Schwarz *et al.*, 2006).

The models are estimated using long-term mean annual loads computed from monitoring data compiled at 425 stations located on the E2RF1 stream network. The mean load estimates are based on water-quality observations collected over the period 1975-1995, and daily streamflow data from the period 1975-2000. The mean load estimates are detrended to the base year 1992 using methods described in Schwarz *et al.* (2006). Each observation in the TN model is weighted to account for variations in the precision of the mean load estimate (procedure described in Alexander *et al.*, 2008, p. S-12); all observations in the TP model receive an equal weight. The land-to-water delivery variables are expressed as differences from their mean, a transformation that aids in the interpretation of the source coefficients, and will enable the comparison of the national models with the regional models described in the next section. Based on physical considerations, all source and aquatic delivery coefficients are constrained to be nonnegative. A complete description of the TN and TP models, including references to data sources and details on the methods used to create the dependent and explanatory variables, is provided in Alexander *et al.* (2008).

## Evaluation of National Nutrient Models for Regional Effects

A coarse assessment of regional effects in the national nutrient models can be made by visual inspection of the national model residuals (Alexander *et al.*, 2008), displayed in Figure 1. The residuals are computed in natural logarithm space, the space in which the SPARROW models are estimated, and for TN are weighted to account for variations in the precision of the load estimate (the observational weights noted above and described in Alexander *et al.*, 2008). A negative residual implies the SPARROW model overpredicts measured load. The most notable feature, evident with both the TN and TP residuals, is the near absence of large residuals in the East. The SPARROW models are considerably less precise in arid environments, notably along the large climate gradient identified with the 100th meridian where large positive and negative errors for both nutrients are prominent. Regional bias is also evident, particularly for TP, which shows a preponderance of large positive residuals in the Northwest region and large negative residuals in the Southwest region. Although much less noticeable, the pattern of regional bias is similar for TN, with a prevalence of positive residuals in the Northwest region and negative residuals in the Southwest region. Also discernable are residual patterns within the regions. In the East region, TN shows a clustering of negative residuals along the South Atlantic coast (a pattern that was previously noted by Hoos and McMahon, 2009), and a grouping of positive residuals in Illinois/Iowa. A prevalence of positive TN residuals is also observed along the Pacific coast, in both the Northwest and Southwest regions. Within-region patterns are more apparent with TP. A cluster of negative residuals is evident in the Lower Great Lakes (within the East region), along the Gulf coast (in both the East and Southwest regions), and the area extending across central Texas, eastern New Mexico, and western Oklahoma (Southwest region). Clusters of positive TP residuals are observed in the East region in southern Florida, through much of the Midwest (in both the East and Northwest regions), and the eastern portions of the Southwest region. The existence of these intra-regional residual patterns underscores the coarseness of the three-region characterization of regional specificity adopted in this study.

A statistical assessment of regional patterns can be made by evaluating the national model residuals for spatial correlation. One such statistic that has been used for this purpose is Moran's *I* (Cliff and Ord, 1973), evaluated here using spatial influence factors applied to the residuals, the factors being the inverse of the distance between paired sites, a weighting scheme that is more sensitive to large-scale regional correlation than to local correlation (Hoos and McMahon, 2009). The Moran's *I* has a statistical significance of <0.001 for both the TN and TP models, providing strong evidence for regional-scale spatial correlation.

To quantify the regional patterns exhibited by the residuals in Figure 1, we developed a fixed-effects regionalization of the national SPARROW models by re-specifying the models to include three region-specific coefficients for each process coefficient. As explained in the Introduction, this approach to regionalization is functionally equivalent to estimating an independent SPARROW model for each region (as long as the regional basins are independent, or if not, as is the case with the East region, part of which is downstream from the Missouri basin component of the Northwest region, as long as region boundaries are located at a monitoring station). A formal statistical evaluation of the effects of regionalization on prediction accuracy was undertaken by comparing measures of fit between the national and regionalized nutrient models. Table 1 presents the difference between the root mean squared error (RMSE) of the national nutrient model and the alternative models with varying degrees of regionalization specified in the model process coefficients. The statistical significance of these differences is evaluated using standard *F*-tests, with the national models representing the null hypothesis of no regionalization. Process coefficients are grouped according to landscape and aquatic properties, with landscape coefficients corresponding to the combined source and land-to-water delivery parameters, and aquatic process coefficients corresponding to the stream and reservoir removal-rate parameters. Separate regional effects are specified for the identified process coefficient groups for either “all regions,” meaning that each region has a unique effect for the stated process coefficient group, or for an individual region, meaning that only the identified region has a unique effect for the stated group; all other regions and unstated process coefficient groups are restricted so as to have a common effect.

**TABLE 1:** The Reduction in the Root Mean Squared Error (RMSE) of Regional Models Incorporating Varying Degrees of Region-Specific Fixed Effects for Land and Aquatic Processes, as Compared to the 1992 National SPARROW Models for Total Nitrogen (TN) and Total Phosphorus (TP) (Alexander *et al.*, 2008)

Alternative Model Specification		TN		TP	
		
Regional Effects	Process Coefficients for Which Region-Specific Fixed Effects Are Incorporated	Reduction in RMSE Compared to National Model Without Regional Fixed Effects†	Number of Fixed-Effects Coefficients‡	Reduction in RMSE Compared to National Model Without Regional Fixed Effects†	Number of Fixed-Effects Coefficients§
All regions	Land and aquatic	0.055**	54	0.073**	45
	Land only	0.046**	50	0.058**	41
	Aquatic only	0.008*	22	0.033**	19
East region only	Land and aquatic	0.025**	36	0.039**	30
	Land only	0.026**	34	0.037**	28
	Aquatic only	<0.001	20	<0.001	17
Northwest region only	Land and aquatic	0.026**	36	0.044**	30
	Land only	0.018**	34	0.024**	28
	Aquatic only	0.007*	20	0.034**	17
Southwest region only	Land and aquatic	0.035**	36	0.053**	30
	Land only	0.032**	34	0.041**	28
	Aquatic only	0.007*	20	0.029**	17

Notes: Statistical significance of a reduction is determined using an *F*-test, with

** denoting significance at the 0.001 level

* denoting significance at the 0.01 level; RMSE reductions without asterisks are not significant at the 0.05 level.

† Calculated as the national model RMSE minus the regional fixed-effects model RMSE. Reduction in RMSE of the natural log residuals can be interpreted as the approximate reduction in the standard error of a prediction of load for a reach, expressed as a share of the predicted load in the reach.

‡ The national model for TN has 18 coefficients.

§ The national model for TP has 15 coefficients.

The results of these tests, as presented in Table 1, show that the national model specification, for either TN or TP, is strongly rejected in favor of fully regionalized models involving both land and water process coefficients jointly, or with land and water processes coefficients regionalized separately. As the reduction in the RMSE, multiplied by 100, can be interpreted as the approximate reduction in the standard error of a prediction of load for any given reach, expressed as a percent of predicted load, the results imply a fully regionalized model improves predictions by 6% for TN and by 7% for TP. Comparisons with models having only single region effects for combined land and water processes (the first line of each group in Table 1, excluding the first group) show rejections of the national model at a 0.001 significance level for all cases, with reductions in RMSE ranging from 3 to 4% for TN and 4 to 5% for TP.

The comparative reductions in RMSE between TN and TP are in general agreement with Figure 1, which shows a more pronounced regional pattern for TP than for TN. The meager improvements in RMSE obtained with the regional fixed-effects model, for both TN and TP, is partly a consequence of the coarse, three-region delineation of the national model used in this analysis. As shown in Figure 1, the regions lack uniformity with respect to the residuals and it may be possible to explain more variation by modifying the delineation of regions to isolate groups of residuals having similar values. However, achieving a large reduction in RMSE by partitioning the U.S. into more regions, delineated on the basis of the residuals depicted in Figure 1, is not guaranteed because SPARROW does not include a constant intercept (Schwarz *et al.*, 2006); any adjustment in a coefficient to correct regional bias must interact with a spatially varying land or aquatic causal variable, the spatial homogeneity of which is not necessarily coincident with that of the residuals.

The significance of comparisons between the national models and individual region fixed-effects models with transport process groups considered separately is also shown in Table 1 (the second and third lines of each group). These results show that land transport processes generally exhibit more region-specific variation than aquatic processes. The reduction in error with regionalization applied to land processes alone across all regions is greater than the reduction for aquatic process regionalization, with this difference being relatively smaller for TP (for TN, a 5% reduction due to land processes *vs.* a 1% reduction due to aquatic processes; for TP, a 6% reduction for land processes *vs.* a 3% reduction for aquatic processes). Land processes are statistically significant, at the 0.05 level, in all individual regions for both TN and TP. Conversely, TN and TP aquatic processes are statistically significant at the 0.05 level only for the individual Northwest and Southwest regions. Nutrient land processes for individual regions generally have a larger effect on RMSE than aquatic processes in all regions, except for TP in the Northwest region.

The statistical significance of the Moran's *I* statistic for the residuals based on the complete regionalization of model processes is 0.144 for TN and 0.023 for TP (see Table 2), indicating that, at least for TN, the three-region delineation of the national model has successfully removed spatial correlation that is sensitive to an inverse distance spatial influence factor. The more pronounced intra-regional patterns in the TP residuals, noted in Figure 1, suggest that removal of spatial correlation requires a partitioning of the U.S. into more than three regions, although such an approach may not be successful for the reason explained above that each SPARROW coefficient must interact with a spatially varying land or aquatic causal variable.

**TABLE 2:** Summary Statistics for the National, Regional Fixed-Effects, and Hybrid SPARROW Models for Total Nitrogen (TN) and Total Phosphorus (TP)

	TN			TP		
		
	National Model	Regional Fixed-Effects Model	Hybrid Model	National Model	Regional Fixed-Effects Model	Hybrid Model
Number of observations	425	425	425	425	425	425
Number of coefficients	18	54	54	15	45	45
Number of coefficients with binding physical constraints	0	5	4	0	1	1
Number of cross-region constraints	0	0	22	0	0	28
Error degrees of freedom	407	376	397	410	381	409
Percent of coefficients with *p* ≤ 0.05*	72	57	76	80	41	80
Average coefficient of variation†	0.403	0.891	0.549	0.369	2.416	0.389
Sum of squared errors (SSE)	124.3	93.3	101.3	238.1	180.9	215.8
Mean squared error (MSE)	0.305	0.248	0.255	0.581	0.475	0.528
Root mean squared error (RMSE)	0.553	0.498	0.505	0.762	0.689	0.726
*R*^2^	0.933	0.953	0.945	0.870	0.902	0.883
Adjusted *R*^2^	0.930	0.947	0.942	0.866	0.891	0.878
Yield *R*^2‡^	0.866	0.906	0.891	0.684	0.760	0.714
Median absolute pct. prediction error§	42.3	40.2	38.4	67.1	52.6	59.1
Significance of Moran's *I*	<0.001	0.144	0.026	<0.001	0.023	<0.001

* For the calculation of the percent of coefficients with *p* ≤ 0.05, a one-tailed *p*-value is used for coefficients subject to physical bounds (all source and aquatic removal coefficients); otherwise, a two-tailed *p*-value is used.

† The standard error of a coefficient estimate divided by its estimated value, averaged over all coefficients in the model.

‡ Yield *R*^2^ adjusts the *R*^2^ to account for the area of each observation's upstream basin, thereby removing from *R*^2^ the inflating effects of a wide range of basin scales (Schwarz *et al.*, 2006).

§ Median absolute error in prediction, expressed as a percent of the monitored load, for 678 TN and 865 TP stations not used in the estimation of the national, regional fixed-effects, or hybrid models.

## Description of the Hybrid Model

The principal conclusion to be drawn from the previous section is that regional variation in the process coefficients is empirically confirmed: the model fits of the regional fixed-effects nutrient models are significantly improved, statistically, relative to those of the national models, although the quantitative improvements are not substantial. The emphasis now turns to regionalizing the national model in such a way that regional coefficients are estimated as precisely as possible without a large compromise in model precision. This section describes a method for regionalizing a national model using the fixed-effects approach by including in the specification a set of cross-region constraints on the fixed-effects coefficients. The resulting model, called a hybrid model, is intended to have more precise coefficient estimates than the regional fixed-effects model, but also reflect regional variation in targeted coefficients sufficient to exhibit improved model fit as compared to the national model.

**FIGURE 2:**
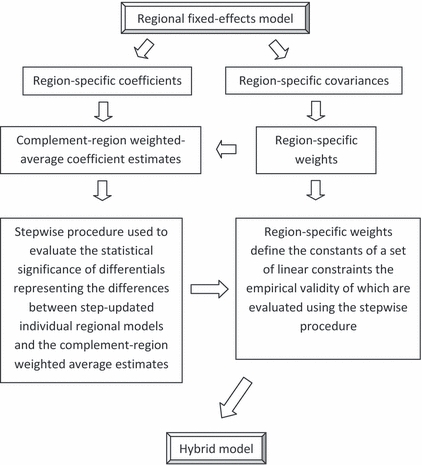
Flow Diagram Describing the Intermediate Steps Applied to the Results of the Regional Fixed-Effects Model to Obtain the Hybrid Model.

Figure 2 summarizes the relationships linking the regional fixed-effects model with the hybrid model. There are three principal steps required to derive the hybrid model: the identification of a set of potential cross-region linear constraints, a statistical evaluation to determine which of these constraints are valid, and the estimation of the fixed-effects model with the valid constraints imposed. As shown in Figure 2, the covariances of the regional fixed-effects model coefficient estimates are used to define a set of weights, these weights forming the constant terms of the potential linear constraints. The weights are also used, in conjunction with the regional model fixed-effects coefficient estimates, to form a set of complement-region weighted-average estimates, a separate average for each fixed-effect coefficient. A stepwise procedure is used to compare each fixed-effect coefficient with its complement-region weighted average counterpart, and the outcome of that comparison determines which of the potential linear constraints are applied in the estimation of the hybrid model. The following discussion explains in detail the motivation underlying each of these relationships.

The imposition of constraints on the regional fixed-effects coefficients begs a question: What specific form should the set of constraints take? Many alternatives are possible, perhaps the most general of which is the set of all possible “pair-equivalence” constraints that equilibrate the process coefficient in one region to a like-process coefficient in another region (e.g., the atmospheric deposition coefficient for the East region equals the atmospheric deposition coefficient for the Northwest region, etc.). If there are *R* regions, and if the national model has *K* process coefficients, this general set consists of *N*_*c*_ = *K* × *R* × (*R* − 1)/2 different constraints (the *K* coefficients of region 1 equated with the *K* coefficients of each of the *R*− 1 other regions, plus the *K* coefficients of region 2 equated with the *K* coefficients of each of the *R*− 2 other regions excluding region 1, etc.), although not all of these constraints are independent. If all constraints are binding, meaning that all process coefficients in any region are equal to the corresponding like-process coefficients in any other region, the resulting model is equivalent to the national model. The practical difficulty with this general constraint set concerns the computation time required to evaluate all of the constraints. There are 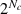 unique combinations of the *N*_*c*_ possible constraints, and the evaluation of each combination requires a separate estimation of the *K* × *R* coefficients of a constrained nonlinear SPARROW fixed-effects model.

An alternative approach is to adopt the perspective of a regional water-quality manager tasked with the problem of specifying a regional SPARROW model. We suppose that the manager is familiar with the regional fixed-effects SPARROW model estimated for the entire nation and has an interest in using the results from this model to improve the precision of some of the coefficients in the subject-region's model. The only restriction we place on the manager is that only the model for the subject region can be re-estimated; the regional fixed-effects coefficient estimates for the complement regions cannot be altered (ultimately, this restriction is removed in the estimation of the hybrid model, which involves the re-estimation of all fixed-effect coefficients across all regions). The proposed approach is to compare the estimate of a process coefficient in the subject region to a weighted average of the fixed-effect coefficients for the complement regions. If the subject-region's estimate is not statistically different from the weighted-average estimate, the weighted-average estimate is substituted for the subject-region's estimate, effectively constraining the subject-region's coefficient to equal the weighted-average estimate; otherwise, the subject-region's coefficient remains a free parameter to be estimated in the subject-region model.

Of practical concern is the determination of the weights used to form the weighted average of the complement-region fixed-effect coefficient estimates. In the Appendix it is shown that there exists a unique set of weights, derived from the covariance matrices of the complement-region fixed-effect coefficient estimates, that minimizes the variance of the difference between the subject-region coefficient estimate and the associated complement-region weighted-average estimate. These weights have a number of interesting features and statistical implications. First, the minimization of the variance of the differential implies that the power of the statistical test comparing the subject-region coefficient estimate to the corresponding weighted-average estimate is maximized, thereby improving the probability of detecting a statistically significant difference in the subject-region's coefficient estimate. Thus, the specified weights serve to minimize the number of constraints imposed in both the subject-region model and, as explained below, the hybrid model.

Second, because the covariance matrices typically include nonzero covariances for all pairings of the process coefficients, the optimal weights are nonzero for *all* complement-region coefficients, not just for like-process coefficients in these regions. Moreover, although the weights sum to one and are necessarily positive for like-process coefficients in the complement regions, the weights may be either positive or negative for dissimilar-process coefficients, depending in a complicated way on the signs and values of the individual elements of the covariance matrices. Thus, for example, in the determination of the complement-region weighted average for the atmospheric deposition coefficient in the East region, the weights will be positive for the atmospheric deposition coefficients in the Northwest and Southwest regions, but could be either positive or negative for, say, the reservoir removal rate coefficients in those regions. As shown in the Appendix, the weights for dissimilar-process coefficients are all zero only if the covariance matrices across all complement regions are identical up to a scaling factor equal to the number of water-quality monitoring sites in the region. Under such conditions, the optimal weights for like-process coefficients are equal to the share of monitoring sites in each region.

A third interesting feature of the optimal weights, also derived in the Appendix, concerns the intuitive interpretation of the weighted-average estimates if the underlying regional model is linear in the coefficients. In this case, the weighted-average estimates reduce identically to the estimates that would result if all like-process coefficients in the complement regions are constrained to have the same value, these values being estimated by weighted least squares with weights equal to the inverse of the variance of the model residuals in each region. Thus, in a linear model, the complement-region weighted-average estimate essentially loses all regional specificity, and the comparison of the subject-region coefficient to the complement-regions’ weighted average is simply a comparison of two regional estimates – the subject-region estimate and the corresponding estimate for the agglomerated complement regions. This equivalence fails to hold for a nonlinear model such as SPARROW because the covariance matrices are themselves functions of the regionally varying process coefficients, implying regional specificity is present at a more fundamental level and cannot be removed. However, in considering the somewhat counter-intuitive features of the optimal weights, as described above, it is useful to understand a context in which the weights make intuitive sense.

The specification of the subject-region model can be achieved by implementing a stepwise procedure. As an initial step, differentials are computed as the differences between the subject-region coefficients and their respective complement-region weighted-average estimates. Each differential is then divided by its respective standard errors (see the Appendix for a derivation of the covariance matrix for differentials) to obtain a differential *t*-statistic, the distribution of which is known to be standard normal in large samples under the assumptions that each region's residuals are independent and identically distributed and that the true differential is zero. If the *p*-value of the differential having the smallest absolute value *t*-statistic is less than a predetermined significance threshold (say, 0.05), then all differentials are presumed to be statistically significant and the stepwise procedure is terminated with the result that none of the potential constraints associated with the region are imposed in the hybrid model. Conversely, if the minimum absolute value *t*-statistic is not statistically significant, the corresponding process coefficient is constrained to equal its weighted-average estimate and the subject-region model is re-estimated. Note that only the subject-region model is re-estimated, the complement-region fixed-effects estimates and implied weighted averages are not altered. It is not necessary to revise the complement-region fixed-effects estimates because these estimates are statistically consistent, meaning that they approach their true values as sample size becomes large, regardless of the cross-region constraints present in the regional fixed-effects model. Revised differentials are computed by differencing the re-estimated subject-region coefficients from their respective complement-region weighted-average estimates, the same weighted averages that were used in the previous step. Standard errors of the revised differentials are derived using the methods developed by Murphy and Topel (1985) (see the Appendix and Supporting Information for a derivation of their method as it pertains to the present application) to account for uncertainty in the value of the constrained coefficient, the coefficient that is set equal to the complement-region weighted-average estimate. The revised differentials are normalized by their respective standard errors and the resulting *t*-statistics of the unconstrained coefficients are ranked to determine the least statistically significant differential. If the least statistically significant differential has a *p*-value below the significance threshold, then all remaining differentials are statistically significant and the stepwise procedure is terminated without imposing any additional constraints; otherwise, the subject-region coefficient corresponding to the least statistically significant differential is set equal to the respective complement-region weighted-average estimate and the stepwise procedure is recycled through another iteration.

The only exception to the stepwise procedure described above concerns cases in which the complement-region weighted-average estimate is inconsistent with physical constraints imposed on the SPARROW model (all source and aquatic removal coefficients are constrained to be nonnegative). Invalid weighted-average estimates are possible because nonzero weights are applied to all complement-region coefficients, not just to like-process coefficients. In such cases, the subject-region coefficient is not constrained to equal the corresponding (invalid) weighted-average estimate, even if the associated differential is not statistically significant.

At the conclusion of the stepwise procedure, the water manager has determined a re-specified model for the subject region, a model that incorporates complement-region information into the analysis by constraining specific subject-region coefficients to equal complement-region weighted-average estimates. Our interest with this model, at least with regard to the development of a hybrid model, is not the particular subject-region estimates; rather, our interest is in the particular constraints that are identified. Each constraint represents an empirically valid linear restriction on the coefficients of the entire regional fixed-effects model, the constants of these constraints being the weights associated with the weighted-average estimates.

In form, the constraints derived from the “water manager's perspective” are more complicated than the relatively simple pair-equivalence constraints described above for the most general constraint system. Collectively, however, the two systems of constraints are equivalent in terms of potential restrictiveness. As shown in the Appendix, if all coefficients, across all regions, are re-estimated to conform to the constraints implied by all the weights across all regions, the fact that the weights sum to one is sufficient to cause all like-process fixed-effects coefficients to be equal, making the model equivalent to the national model. Thus, the set of all possible constraints implied by the weighted averages exactly duplicates the most restrictive collection of pair-equivalence constraints previously described.

Duplication of the stepwise procedure over all *R* regions provides a complete set of constraints. However, as shown in the Appendix, *K* of the differentials, across all regions, are linearly dependent, implying *K* of the hypothesis tests undertaken in the collective stepwise procedures are not independent. Moreover, a logical inconsistency may exist in the results of the stepwise procedure applied to all regions. To see this, suppose there is only one process coefficient in a model consisting of three regions, implying there are three fixed-effects coefficients to be estimated and three potential constraints to evaluate using the stepwise procedure, one constraint for each region. If the stepwise procedure identifies the constraint is binding in the first region and, in the second region, the constraint is not binding, then an inconsistency arises if the constraint in the third region is determined to be binding. Due to the dependence across constraints, if two constraints are binding then the third must also be binding. Unfortunately, there is no guarantee that application of the stepwise procedure to all regions identifies a consistent set of constraints.

In the case of inconsistency across constraints, it is arbitrary whether to accept all identified constraints. Moreover, with multiple process coefficients it is difficult to know whether inconsistency is present. One way to guarantee there are no inconsistencies in the accepted constraints, which also implies that all imposed constraints are independent, is to exclude one of the regions from the stepwise procedure. The exclusion of a region possibly leads to too few constraints; however, too few constraints may be preferable to imposing too many, and is consistent with the general objective of minimizing the number of constraints applied in the hybrid model (see the above discussion pertaining to the use of optimal weights).

The perspective adopted above, whereby water managers specify individual regional models that make use of extra-regional information, provides a feasible approach to identifying the constraints used in the specification of the hybrid model. Given the estimates from the unrestricted regional fixed-effects model, each regional manager uses a stepwise procedure to evaluate at most *K* possible constraints. Moreover, the evaluation of each constraint requires the estimation of a relatively simple regional model consisting of only *K* coefficients. Duplication of this effort over *R*− 1 regions provides a complete set of constraints, implying the total number of evaluated constraints is no more than *K* × (*R*− 1). If there are many regions (*R* ≥ 3), this number is considerably less onerous than that for the general pair-equivalence constraint set noted above.

As a final step, the empirically validated constraints described above are imposed in the re-estimation of the regional fixed-effects model to produce the hybrid model. The assumed properties of the SPARROW model residuals imply the covariances of the coefficient estimates are consistently estimated. By a well-known theorem (Amemiya, 1985; theorem 3.2.6), this implies the constants of the linear constraints are also consistently estimated and, with large samples, there is no statistical advantage to re-estimating them in light of the constraints identified by the stepwise process. The constraints imply specific coefficients in the hybrid model are restricted to equal a weighted average of hybrid model coefficient estimates for the complement regions. Although the weights are identical to those produced from the original regional fixed-effects model, the weighted averages are not, the coefficients to which the weights are applied having taken on the new values obtained through the simultaneous estimation of all coefficients in the hybrid model. Note, also, that due to the nature of the weights (see above and the Appendix), any constraint includes coefficients from all regions of the model. This means information from every region is used in the estimation of any individual coefficient. Like the coefficients of the regional fixed-effects model, hybrid model coefficients are consistent. The imposition of the constraints limits the regional variation in hybrid model coefficients but also reduces the standard error of their estimates, the estimation of which requires a special procedure (Gallant, 1987; see the derivation of the standard errors of the hybrid model in the Appendix and Supporting Information) to account for the constraints.

In summary (refer to Figure 2), the hybrid model is a linearly constrained version of the regional fixed-effects model. The coefficient covariances from the regional fixed-effects model are used to compute a set of weights, these weights representing the constants of potential linear constraints to be applied in the analysis. To determine which of the potential constraints are actually imposed, the weights are combined with the coefficient estimates from the regional fixed-effects model to obtain a complement-region, weighted-average estimate for each fixed-effects coefficient in the model. A stepwise procedure is used to identify which fixed-effects coefficients are significantly different from their associated complement-region weighted average. The specific constraints applied in the estimation of the hybrid model are those associated with weighted averages that are not significantly different from a fixed-effects coefficient.

## Evaluation of Results of the Hybrid Models for Total Nitrogen and Total Phosphorus

As explained in the Introduction, the precision of the estimated coefficients of the regional fixed-effects model can be improved, with a corresponding loss in model fit, by imposing cross-region constraints on the regional fixed-effects coefficients, effectively expanding the scale of the analysis beyond a strictly regional scale. To demonstrate the tradeoff between coefficient precision and model precision, the methodology of the hybrid model described in the preceding section is applied to the regional fixed-effects model estimated for the three regions shown in Figure 1.

The stepwise procedure was used to identify which of the fixed-effects coefficients to constrain to equal a linear function of the complement-region fixed-effect coefficients, the parameters of the linear function being the optimal weights computed from the regional fixed-effects model. As explained in the previous section (and shown in the Appendix), the set of all possible constraints is not mutually independent, and an independent and consistent set of constraints can be obtained by excluding one of the regions from the stepwise procedure. Even though the selection of which region to exclude is arbitrary, the results of the analysis are not invariant to which region is chosen. The East region has the most observations (see Figure 1), making this region the likely source of regionally specific coefficients and, consequently, most likely the source of the fewest constraints. Therefore, the East region was excluded from the stepwise procedure. Note that the exclusion of the East region from the stepwise procedure does not mean that the fixed-effect coefficients of this region are excluded from the overall set of constraints applied in the hybrid model. As remarked above, each applied constraint includes every fixed-effect coefficient in the model, including the region excluded from the stepwise procedure. The inclusion of the East region coefficients in each imposed constraint implies that the hybrid model estimates of these coefficients will differ from their values obtained with the regional fixed-effects model.

To evaluate the validity of the *t*-tests used in the stepwise procedure, the residuals from the regional fixed-effects model for the Northwest and Southwest regions, the only regions subjected to the stepwise procedure, were assessed for independence and homoscedasticity (the statistical property that residuals of the model have a common variance). For both regions, for both TN and TP, the Moran's *I* was found to be statistically insignificant, the smallest *p*-value among the four statistics being 0.38, a value that is not even marginally significant. The assumption of homoscedasticity of the residuals was evaluated using a variant of a test for heteroscedasticity developed by White (1980), whereby the squared residuals (for TN, the squared residuals are multiplied by the observational weights) of the regional fixed-effects model are regressed on the derivatives of the model predictions at each monitored reach with respect to each model coefficient (i.e., the gradients of the nonlinear SPARROW model – the analog of the explanatory variables in a linear model). In all cases, the *F*-statistics for these regressions were not significant at the 0.05 level, indicating that heteroscedasticity was not a concern.

The stepwise procedure was used to identify 22 constraints, each constraint being applied to the 54 fixed-effects coefficients (41% of the coefficients were constrained) included in the TN model. The remaining 32 potential constraints were not applied either because they pertain to potentially nonindependent constraints associated with the East region (18 coefficients), were not constrained due to the corresponding weighted-average estimate being inconsistent with physical bounds that all source and aquatic removal rate coefficients be nonnegative (2 coefficients in the Southwest region), or had statistically significant regional differentials (12 coefficients, 7 in the Northwest region and 5 in the Southwest region). Coefficients that were unconstrained, aside from all coefficients in the East region, were: wheat and pasture/range nitrogen application, barren land, artificial drains, and stream removal rate (both the Northwest and Southwest regions), corn/soybean nitrogen application and temperature (Northwest region), and urban population and soil permeability (Southwest region). For the TP model, the stepwise procedure identified 28 constraints to be applied to the 45 fixed-effects coefficients (62%), consisting of all coefficients for both the Northwest and Southwest regions, except the Southwest region values of the specific catchment area and stream removal rate coefficients, both of which had statistically significant differentials.

The TN and TP regional fixed-effects models were re-estimated with nonnegative physical bounds placed on all source and aquatic removal rate coefficients, and with the 22 and 28 cross-region linear constraints identified from the stepwise process. Statistical summaries of the resulting estimations of the hybrid models, and their comparison to statistics for the national and regional fixed-effects model without cross-region constraints, are given in Table 2. In terms of percent of coefficients that are statistically significant at the 0.05 level, the hybrid models perform much better than the regional fixed-effects models, for both TN and TP – a consequence of the cross-region constraints, which greatly increase the error degrees of freedom (number of observations, plus number of constraints, minus number of estimated coefficients) of the hybrid model as compared to the regional model. The TN hybrid model average coefficient of variation (the ratio of each coefficient's standard error to its estimated absolute value, averaged over all coefficients in the model), a normalized measure of the imprecision of all the coefficients in the model, is about 38% [100 (0.891 − 0.549)/0.891] lower than that for the regional fixed-effects model. The hybrid model improvement in coefficient precision is even greater for TP, with an average coefficient of variation that is 84% [100 (2.416 − 0.389)/2.416] lower than that for the regional fixed-effects model.

The reduced model fit caused by the imposition of constraints to obtain the hybrid model can be measured by a number of statistics, including the RMSE, *R*^2^, and yield *R*^2^. As the model is estimated in logarithmic space, 100 times the RMSE can be interpreted as the approximate standard error of a predicted load for any arbitrary reach in the model, expressed as a percent of the predicted load (this approximation is derived from a first-order Taylor expansion of the error in load, expressed in real space; see Schwarz *et al.*, 2006, for a related discussion). The *R*^2^ and yield *R*^2^ are computed from load and predicted load having a logarithm transformation. The yield *R*^2^– the *R*^2^ computed using predicted and observed values of the logarithm of load divided by upstream basin area – is a measure of fit that effectively removes from *R*^2^ the inflating effects from estimating a model that encompasses a wide range of basin scales (Schwarz *et al.*, 2006).

In terms of RMSE, the TN hybrid model is only slightly less precise than the regional fixed-effects model, the difference in RMSE between the hybrid and regional fixed-effects models being only 0.007 (0.505 − 0.498). Thus, using the approximation described above relating differences in RMSE to differences in the standard error of load, expressed as a percent of load, the predicted TN load for the hybrid model has a standard error that is only 0.7% greater than that of the regional fixed-effects model and nearly 4.8% [100 (0.505 − 0.553)] lower than the national model. Similarly, the *R*^2^ and yield *R*^2^ are nearly equal between the regional fixed-effects and hybrid TN models, the difference being only 0.008 for *R*^2^ and 0.015 for yield *R*^2^.

The TP hybrid model has relatively more constraints applied than the TN model, implying the sacrifice in model fit as compared to the regional fixed-effects model is greater. The RMSE of the hybrid model is approximately halfway between the national model and the regional fixed-effects model, with the standard error of predicted load, expressed as a percent of load, being approximately 3.7% [100 (0.726 − 0.689)] greater than that for the regional fixed-effects model and 3.6% [100 (0.726 − 0.762)] less than that for the national model. The *R*^2^ and yield *R*^2^ of the TP hybrid model are only 0.019 and 0.046 less than those for the regional fixed-effects model.

The relative model fits of the hybrid and regional fixed-effects models can also be evaluated in terms of an *F*-test applied to the sum of squared errors and error degrees of freedom statistics reported in Table 2. For TN, the *p*-value of the *F*-statistic evaluating the statistical significance of the difference in sum of squared errors between the two models is 0.061, indicating that the model fit of the regional fixed-effects model is not significantly better than the hybrid model. The *F*-test for the TP model gives a *p*-value <0.001, implying the regional fixed-effects model has a significantly better fit than the hybrid model.

Perhaps a more useful measure of model performance is prediction accuracy. Such an assessment includes not just model error (as expressed by the MSE), but also the sample error associated with uncertainty in the coefficient estimates. To assess model accuracy we compiled concentration and flow data (see Saad *et al.*, this issue) to compute mean loads, detrended to a base year of 1992 (see the load estimation method described in Alexander *et al.*, 2008), for an additional 678 (TN) and 865 (TP) stations linked to the E2RF1 reach network but not used in the estimation of any of the models described in this article. Predictions from the national, regional fixed-effects, and hybrid models were used to compute error as a percent of the monitored mean load values at the reach locations of the additional stations (see Schwarz *et al.*, 2006, for a description of the prediction methodology). Table 2 reports the median absolute value of the percent prediction error for TN and TP across all stations for each model. The results are very similar to those obtained for RMSE. For TN, the three models have nearly the same accuracy, with the national model being least accurate (median absolute prediction error of 42.3%) and the hybrid model having slightly better accuracy (38.4% absolute error) than the regional fixed-effects model (40.2% absolute error). Predictions from the TP models are less accurate than the TN models, with the national TP model having the least accuracy (67.1% absolute error) and the regional fixed-effects model the greatest accuracy (52.6% absolute error). As with RMSE, the TP hybrid model has a median absolute error (59.1%) that is approximately halfway between the national and regional fixed-effects models.

The Moran's *I* statistic, derived from the residuals of the hybrid model and reported in Table 2, is statistically significant for both TN and TP, indicating the presence of spatial correlation in these models. Given the detection of spatial correlation in the regional fixed-effects model, this result is not unexpected for TP. The presence of spatial correlation for the TN hybrid model, however, suggests that the constraints applied to obtain the TN hybrid model have induced a regional pattern in the residuals.

**FIGURE 3:**
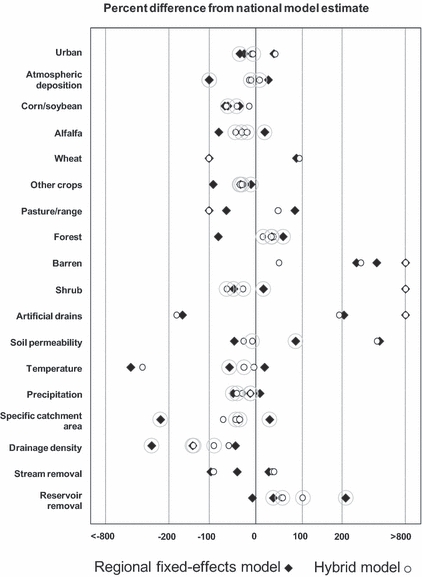
A Comparison of Regional Fixed-Effects and Hybrid Model Estimates for Total Nitrogen (TN). Models are based on data from Alexander *et al.* (2008). Plotted values represent the coefficient estimate expressed as a percent difference from the national model estimate (see Alexander *et al.*, 2008, for the national model coefficient estimates). Circled values pertain to regional fixed-effect and hybrid model coefficients that are constrained in the stepwise procedure and consequently generate a constraint in the estimation of the hybrid model. Note that the horizontal scale is compressed in both the right and left margins, so much so that values on the left and rightmost reference lines are unresolved.

**FIGURE 4:**
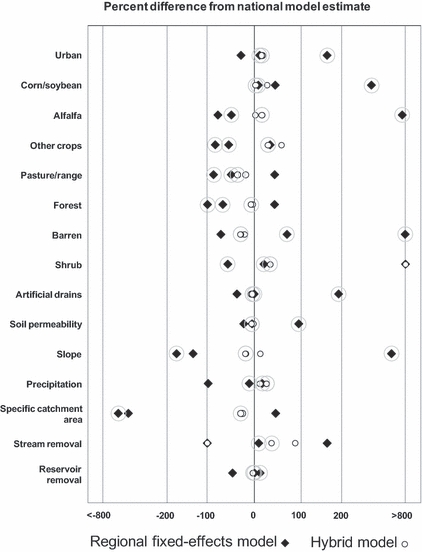
A Comparison of Regional Fixed-Effects and Hybrid Model Estimates for Total Phosphorus (TP). Models are based on data from Alexander *et al.* (2008). Plotted values represent the coefficient estimate expressed as a percent difference from the national model estimate (see Alexander *et al.*, 2008, for the national model coefficient estimates). Circled values pertain to regional fixed-effect and hybrid model coefficients that are constrained in the stepwise procedure and consequently generate a constraint in the estimation of the hybrid model. Note that the horizontal scale is compressed in both the right and left margins, so much so that values on the left and rightmost reference lines are unresolved.

The coefficient estimates, standard errors and *t*-statistics for the TN and TP hybrid models, along with detailed results for the national and regional fixed-effects models are reported in the Supporting Information. Figures 3 and 4 summarize these results in a way that allows for a visual comparison of the estimates. The figures display the coefficient estimates for the hybrid and regional fixed-effects models expressed as a percent difference from the national model estimates. Thus, a value of zero on the graph implies the coefficient equals the national model estimate, and hybrid and regional model estimates that have a binding physical constraint (zero values for source and aquatic removal rate coefficients constrained to be nonnegative) are plotted on the −100% difference reference line (physical constraints imply no hybrid or regional model difference estimates are less than −100% for any source or aquatic removal process coefficient). Although not readily apparent in all cases, each process coefficient includes three plotted values, one value for each region, for each of the hybrid and regional fixed-effects models. Circled values pertain to hybrid and regional model coefficients that are identified in the stepwise procedure to be constrained to equal a weighted average of the complement-region coefficient estimates; thus, hybrid model values that are not circled are unconstrained. In order to display all the coefficient estimates in a single graph it is necessary to use a nonlinear scale, one that greatly compresses the horizontal scale in the positive and negative tails of the figure, so much so that values less than −800% or >800%, plotted along the left and rightmost reference lines, are effectively unresolved. For TN, values along the rightmost reference line range from 1,499% (hybrid model artificial drains estimate in the Southwest region) to 4,760% (hybrid model barren estimate in the Northwest region); for TP, values near or along the rightmost reference line range from 773% (regional fixed-effects model barren estimate in the Southwest region) to 2,402% (regional fixed-effects model shrub estimate in the Northwest region).

A comparison of the various model coefficient estimates for TN is shown in Figure 3. Generally, the range of regional variability differs considerably across the process coefficients, with the national model providing a representative estimate for each coefficient. As expected, the greatest range of variation, for most process coefficients, is exhibited by the regional fixed-effects model estimates. Large (absolute value >200%) differences from the national estimates are observed for the regional fixed-effects estimates of a number of process coefficients, including: barren and shrub lands, artificial drains, soil permeability, temperature, specific catchment area, drainage density, and reservoir removal. Variation in the hybrid model estimates approximates the variation in the regional fixed-effects estimates for a few process coefficients (wheat, pasture/range, barren and shrub lands, artificial drains, soil permeability, temperature, and stream removal); generally, however, the hybrid model estimates are closer to the national model estimates than are the regional fixed-effects estimates. Two hybrid model coefficients that represent the largest differences from the national model estimate, shrub land (East region) and barren land (Northwest region), correspond to the smallest sources in their respective regions (Alexander *et al.*, 2008), so the effect of the large coefficients on predicted load is relatively minor. Using ±100% difference from the national model estimate as a benchmark for a substantial regional variation, there are 10 such coefficients (19% of the 54 coefficients) exceeding this threshold for the TN hybrid model. Hybrid model estimates subject to a cross-region constraint (i.e., hybrid estimates that are constrained to equal a weighted average of the complement regions’ hybrid model estimates; values that are circled in the figure) are generally within 100% of the national model estimates, except for the drainage density and reservoir removal estimates.

A comparison of coefficient estimates between the national, regional fixed-effects, and hybrid TP models is shown in Figure 4. As with TN, the national model provides a representative estimate for each coefficient. Unlike TN, the TP regional fixed-effects model estimates show greater variation than the hybrid model estimates for all process coefficients. The variation in the hybrid model estimates approximates that of the regional fixed-effects estimates only for shrub land and stream removal, the latter case being an example where both the regional fixed-effects and hybrid model estimates face a binding physical constraint (both estimates are at plotting position −100%, implying they are evaluated at zero – the physical bound placed on aquatic removal rate coefficients). Many of the most extreme regional fixed-effects estimates in Figure 4 appear spurious, their values being constrained in the hybrid model estimation (circled values). Only the coefficient for shrub land has a large deviation from the national model and is not subsequently constrained in the hybrid model estimation, although this value is unconstrained because it pertains to the East region, the region excluded from the stepwise process. As remarked above, shrub land pertains to an insignificant source in the East, so the effect of the coefficient on predicted load is negligible. All hybrid model estimates subject to a cross-region constraint (the circled values) lie within ±100% of the national model estimate, which implies that many of the extreme regional fixed-effects estimates become “controlled” by the hybrid model methodology. If the benchmark for a significant regional variation is an absolute difference greater than 100% from the national model estimate, the TP hybrid model has only one such coefficient (the shrub land coefficient for the East region; representing only 2% of the 45 total coefficients).

The results of the analysis show the hybrid approach achieves greater precision in the coefficient estimates as compared to the regional fixed-effects model, with only a modest sacrifice in prediction accuracy. The greater precision in the hybrid model coefficients for TN comes at virtually no cost in terms of model fit, whereas the much larger improvement in the precision of the TP hybrid model coefficients requires a sacrifice of approximately half of the model fit advantage of the regional fixed-effects model over the national model. Very few hybrid model coefficients show evidence of large regional variation, the most notable cases involving barren and shrub land in both the TN and TP models. It is possible that the data for these land classes contain classification errors, or the contaminant sources they represent are not sufficiently differentiated, and an improved model specification would be possible by combining the two classes into a single variable. Both the TN and TP hybrid models exhibit spatial correlation in the residuals, a likely byproduct of the coarse regionalization adopted for this study.

## Discussion

An issue frequently raised by the regionalization approach concerns the source of regional variation in the coefficients. From a modeling perspective, it is reasonable to suppose that the causes of regional variation are unrelated to variables that could be employed in the statistical analysis, for if such variables were the cause their use in the model would alleviate the need for regionalization. Consequently, regionalization likely reflects the absence of relevant data in the analysis. For example, the natural phosphate content of basin minerals, a prominent variable affecting TP (García *et al.*, this issue), is absent from the models described here due to a lack of consistent data at the national scale.

Even if pertinent variables are available, regional patterns in the residuals may be evident if the variables are measured with error, or if the functional relation between water quality and the causative variables is too complex to be adequately specified in a large-scale model. An example of a variable measured with error from the TN model is atmospheric deposition, which pertains only to wet deposition and excludes dry deposition. In addition, the surrogate land use variables employed in both the TN and TP models are incomplete measures of nutrient loading for specific land types, and are likely the source of regional coefficient variation. Given the scale of the analysis, therefore, regional variability in the coefficients is expected to be pervasive, making it difficult to attribute a specific cause.

The fixed-effects approach is just one of many that have been used to introduce regional variation into statistical models. An alternative is the “random effects” approach (Judge *et al.*, 1985), also called the hierarchical or multilevel method (Reckhow *et al.*, 2009; Kashuba *et al.*, 2010; Qian *et al.*, 2010), which specifies model coefficients to be random variables having an assumed multivariate probability distribution with unknown parameters. The advantage of this approach is that only the parameters of the multivariate distribution for the coefficients need to be estimated, not the individual effects for each region, the estimation of which could be difficult if there is insufficient within-region variation in basin attributes. The multilevel method also has the potential to provide a richer description of the origin of error in the model, which is useful in the evaluation of prediction uncertainty. However, the method places rather strict assumptions on the statistical distribution of the regional coefficients (Mundlak and Yahav, 1981); typically, the regional coefficients are assumed to be normally or log-normally distributed and independent across regions. Moreover, the method may be difficult to implement computationally in the context of a nonlinear model, such as SPARROW, particularly if the multivariate distribution of the coefficients has a complicated covariance structure.

Another popular approach is the region of influence approach (Acreman and Wiltshire, 1987; Burn, 1990a,b;), which posits a different set of coefficients for each observation in the analysis. The procedure is to develop a unique estimation sample for each observation, one that has explanatory variable characteristics that come closest to the given observation. Similar methods are the regression tree analysis and the cluster analysis (Burn and Boorman, 1993; Robertson *et al.*, 2006; Soranno *et al.*, 2010). A common advantage of these “data-driven” methods is the flexible concept used to define a region: the delineation of a region is based on all model variables, not solely on the spatial dimension. However, this flexibility also makes it difficult to interpret the estimated coefficients and compare them to values obtained in other studies. For this reason, these methods may be better suited for prediction of water-quality characteristics at unmonitored sites; they may be less appropriate for applications requiring precise estimates of individual model coefficients, as is necessary for the evaluation of water-quality response to alternative management or forecasting scenarios.

A common purpose for employing regionalization methods is to account for regional variation in the data even where the causes of the variation are difficult to identify explicitly. The fixed-effects method, like the other methods described in this section, assigns regional variation to specific process coefficients of the model, an assignment that may provide some insight as to the underlying latent biogeochemical or hydrological processes, human activities, or data imperfections driving regional variation (see Hoos and McMahon, 2009, for a discussion of how a causal relationship could be identified in the context of a fixed-effects approach to regionalization). However, the lack of a complete causal understanding for regional variation with these approaches may limit the utility of regionalized models in the generation of model predictions under alternative management scenarios. For example, if regional variation reflects differences in unspecified climate conditions, the regional model may be less useful for simulating the effects on water quality resulting from climate change – without knowledge that the regional effect is due to climate it is not possible to modify this effect for different climate conditions. This issue also has implications for quantifying the uncertainty of simulations based on the model. The inclusion of regional effects may improve model fit, but given the lack of understanding regarding the nature of the regional variation, it remains an open question as to whether this reduced error should translate into lower error in the simulation results.

The fixed-effects approach leaves unanswered a clear and definitive methodology for defining the geographic limits of a region. The methodology imposes a generally arbitrary regional structure, usually defined according to hydrological properties (e.g., watersheds), and then uses statistical evidence to determine the relevance of this structure. Other methods, such as cluster and regression tree analyses and the method of regional regression, offer a more flexible approach that uses characteristics of the data to define regions. However, if the underlying factors causing regional variation are unknown, uncertainties will remain with these approaches as to how reliably to use regional models in simulation applications. Another approach may be to delineate regions based on a preliminary assessment of residuals using a nonregionalized model. As is shown in Figure 1, residual patterns are evident for both TN and TP along the coastal areas of the eastern U.S. and Gulf of Mexico, patterns that do not fit perfectly with the regional basins used for this study. However, as remarked above, each coefficient in a SPARROW model must interact with a spatially varying land or aquatic causal variable, so that regional patterns in the model coefficients need not translate into discernable regional patterns in the residuals, drawing into question the utility of this approach.

The standard approach to regionalization is one in which each regional model is estimated independently (as with the NAWQA MRB regional models included in this Featured Collection). As discussed in the Introduction, the principal disadvantage of this approach is insufficient within-region spatial variation of the explanatory variables, leading to imprecise estimates of the model coefficients. There are, however, several mitigating circumstances acting in favor of such an approach. The independent regional analysis may allow for scaling the level of effort required to develop certain geospatial datasets. For example, the resources required to produce a good point source dataset should scale, at least approximately, with the basin area of the analysis. It may also be possible in a regional analysis to focus resources on the development of certain geospatial datasets that are important for the region of interest but of lesser importance elsewhere. A map showing the location of millponds might be of particular interest for a model pertaining to the Northeast U.S., but of lesser importance for a model focused on the Midwest. The independent regional analysis also allows greater flexibility in the specification of the model, such as the capability to use geospatial data that are available only for the region of interest. This latter point brings to attention one of the weaknesses of the fixed-effects approach, that it does not address different specifications of the model, either in terms of functional forms assumed for the transport processes or the particular contaminant sources and other variables used in the analysis. Different specifications of the model across different regions may be required to address cases in which a source is poorly defined in a specific region leading to a potentially spurious estimate of a model coefficient, such as the cases noted above in the results of the hybrid models for the barren land TN model coefficient in the Northwest region and the TN and TP model shrub land coefficients in the East region. The independent NAWQA MRB models of this Featured Collection demonstrate the utility of incorporating into their analyses factors other than those used in a national analysis. Indeed, differences in these factors, as well as a different time period (the independent regional models use a base year of 2002, compared to the year 1992 used for this study) and a large difference in the number of stations used in model estimation may help explain any discrepancies in model results among this study and the independent MRB models.

The results of this study show that there are likely to be gains in model inference by combining the results of independent regional models with those of national-scale models, a direct consequence of more information being better than less. Perhaps a better incarnation of this maxim, however, is achieved through Bayesian analysis. Under a Bayesian approach, the national-scale model could serve as prior information for the estimation of a regional scale model (Qian *et al.*, 2005; Qian and Reckhow, 2007). Rather than deciding between coefficients based on information from either the national or regional scale, as is done under the fixed-effects approach described in this article, the Bayesian posterior distribution for the regional coefficients would reflect the combined information from both scales.

The method of regionalization employed in this article, whereby the variation of coefficients across space is constrained to improve statistical inference, can also be applied to the analysis of the variation of coefficients across time. In a temporal context, one can imagine multiple SPARROW models, each covering the same study area, estimated independently according to water-quality conditions at different points in time. The pertinent question is whether it is necessary to specify different values for the coefficients for each modeled time period, or whether a given coefficient could be constrained to equal a weighted average of the coefficients for the other periods. Conceivably, the method described here could be used to form cross-period constraints on the coefficient estimates. The method may need to be modified if the assumption that the errors in the coefficient estimates are independent across time is untenable, as would be the case if the residuals of the SPARROW model were serially correlated.

## Summary and Conclusions

The findings of this study demonstrate that regionalization of national SPARROW models for TN and TP using the fixed-effects approach has the potential to provide modest, albeit highly statistically significant improvements of 6 and 7%, respectively, in the accuracy of model predictions. However, this enhanced accuracy comes at a cost of reduced precision of the regional fixed-effects coefficient estimates relative to the national model estimates. A method is proposed to combine national and regional information to expand the variation in the underlying process variables, thereby improving the precision of the regional fixed-effect coefficient estimates. The method is applied to three regions delineating major water basins of the conterminous U.S., leading to the specification of hybrid models for TN and TP. The results of these analyses show that the method is successful in improving the precision of the process coefficient estimates as compared to a standard regionalized fixed-effect approach, while providing modest reductions in the standard error of TN and TP model load predictions, as compared to the national model without regionalization, of 5 and 4%. The hybrid model estimates show little evidence of strong regional variation in the coefficient estimates. Only 19% of the TN hybrid model coefficients and just 2% of the TP hybrid model coefficients have more than ±100% deviation from the national model estimate. This lack of strong regional variation is possibly due to the coarse partitioning of the U.S. into just three regions.

From a theoretical perspective, the regional boundaries used in a regionalization analysis should delineate natural areas having approximately homogeneous values of the underlying model coefficients. As the hybrid model developed for this study constrains the regional variation of *individual* coefficients, all coefficients need not adhere to identical spatial patterns of homogeneity. This also means the method can be useful in limiting the variation of coefficients across regions that do not strictly conform to the natural areas described above, such as the case where the regional analysis requires the delineation of areas along arbitrary political boundaries to accommodate specific management considerations.

The national model residuals for TN and TP display distinctive regional patterns; however, these residual patterns may not be fully indicative of spatial patterns in the underlying values of the model coefficients. Additional work is required to better define regions of the nation having the characteristics of natural areas reflecting homogeneous values for model coefficients. Such an effort will be greatly aided by including the large set of monitoring data compiled as part of the NAWQA MRB studies appearing in this Feature Collection. Those data should allow for the specification of many more regions, resulting in greater improvements in model fit than obtained here. The added observations would also reduce the standard errors of the regional fixed-effects coefficients, or at least moderate the increase in standard errors caused by the larger number of fixed effects associated with a finer regional delineation of the nation. Subsequent analysis should also investigate alternative formulations of the model beyond that of the previously published national models to determine if some of the regional patterns in the hybrid model coefficient estimates documented above are an artifact of model misspecification. Regardless of the specific modifications to the analysis enabled by a larger dataset, the pooling of information across regions, using methods akin to those described in this article, should prove helpful in achieving a favorable tradeoff between precision of the coefficient estimates and precision of the model predictions.

## Appendix

The following presents a mathematical formulation of the hybrid model, beginning with a derivation of the complement-region weighted-average estimates and region-specific coefficient differentials. Let there be *R* regions: the subject region, *s*, and the *R* − 1 complementary regions, indexed by *r*. Define  to be the estimated vector of *K* process coefficients associated with region *r*. The estimated region *s* coefficient differential, , is defined as the difference between the region *s* estimated coefficient vector, , and a weighted average of the estimated coefficient vectors for the complementary regions,

(A1)

where **W**_*s*,*r*_ is a *K* × *K* matrix of weights, with , **I**_*K*_ being the *K*-dimensional identity matrix. Under the assumption that the error terms of the regional models are not spatially correlated across regions, the regional coefficient estimates are independent, having region-specific covariance matrix **V**_*r*_. Thus, the covariance matrix for the region *s* coefficient differential vector is

(A2)

*Proposition 1*: the covariance matrix of the estimated regional differentials is minimized (i.e., the quadratic form  is minimized for arbitrary *K* × 1 vector **x**) if the weights are set according to

(A3)

*Proof*: Consider setting the weights equal to the proposed value, plus some arbitrary matrix **M**_*s*,*r*_, so that

(A4)

Because the weights must sum to **I**_*K*_, it must be the case that , **0**_*K*_ being a *K* × *K* zero matrix. Upon substituting Equation (A4) into Equation (A2), the covariance matrix of the regional differentials becomes

(A5)

As **V**_*r*_ is positive definite,  must be a nonnegative matrix. Thus, any quadratic form derived from Equation (A5) is minimized if and only if . By theorem 12.2.5 of Graybill (1969), the sum of nonnegative matrices is zero if and only if each of the nonnegative matrices comprising the sum are also zero, implying  for all *r*. As **V**_*r*_ is positive definite, there exists a nonsingular matrix **P**_*r*_ such that  (Graybill, 1969, theorem 12.2.2), implying , where **U**_*s*,*r*_ = **M**_*s*,*r*_**P**_*r*_. The *i*th diagonal element of  is given by , which equals zero for each *i* element if and only if *U*_*s*, *ri*, *k*_ = 0 for all *i*, *k*. Thus, **U**_*s*,*r*_ = **M**_*s*,*r*_**P**_*r*_ = **0**_*K*_, which, given that **P**_*r*_ is nonsingular, implies **M**_*s*,*r*_ = **0**_*K*_. Therefore, Equation (A3) is the unique value for the weights that minimizes any quadratic form based on the covariance matrix of the regional coefficient differentials.

With weights set according to Equation (A3), the covariance matrix for , is given by

(A6)

An implication of Equation (A6) being a minimum covariance matrix is that the diagonal elements have the smallest possible value subject to the constraint that the weights sum to one. Consequently, the test for significance of a given regional differential is made as sensitive as possible. Note, also, that the assumption of independent errors across regions implies that the weights minimize both the variances of the differentials and the variances of the complement-region weighted-average estimates.

An examination of some special cases can enhance understanding of the properties of the optimal weights. The optimal weights described above have a straightforward large-sample interpretation if it is assumed that the asymptotic covariance matrix of  is identical for each region, where *N*_*r*_ is the number of monitoring sites in region *r*. In this case,  and the asymptotic optimal weights are simply the share of total observations in each complement region, .

The optimal weights also have a straightforward interpretation if the underlying model is linear. Let the water-quality model for region *r* be given by **y**_*r*_ = **X**_*r*_**β**_*r*_ + **e**_*r*_, where **y**_*r*_ is an *N*_*r*_ × 1 vector of water-quality load measurements, **X**_*r*_ is an *N*_*r*_ × *K* matrix of explanatory variables, and **e**_*r*_ is an *N*_*r*_ × 1 vector of independent, identically distributed residuals having mean zero and variance . In this case, the differential is computed as

(A7)

which is the difference between the ordinary least squares estimate of the process coefficients for the subject region *s* and the weighted linear least squares estimate of the process coefficients for the combined *R* − 1 complementary regions. Other than accounting for differences in the mean squared error (MSE) across regions, the optimal weights result in a test statistic that does not distinguish between individual complementary regions and is equivalent to comparing the ordinary least squares estimate of region *r* to a single like-process coefficient estimated from all complement region data pooled together.

The analysis is more complicated if the underlying model is nonlinear in the coefficients, such as in a SPARROW model. In that case, the regional covariance matrices, **V**_*r*_, are functions of the underlying regional coefficients, **β**_*r*_, implying it is not possible to simply pool all complement region data. Nevertheless, the regional deviation statistic, and its associated covariance matrix given in Equation (A6), provides a useful *t*-statistic for evaluating the significance of regional differences in process coefficients. The nonlinear estimate of  is asymptotically efficient (in the sense of having minimum variance), and the *t*-statistic, computed in conjunction with the associated covariance matrix, itself formed from standard nonlinear coefficient covariances **V**_*j*_ for the individual regions (see, for example, Amemiya, 1985), is asymptotically distributed standard normal as long as the underlying regional coefficients have the same asymptotic properties and sampling of the regions is proportionate to total sample size.

The stepwise procedure applied to subject region *s* requires repeated estimation of the region *s* model, at each step evaluating the statistical significance of the regional differentials. The initial evaluation of the differentials uses the standard errors implied by Equation (A6). Evaluations in subsequent steps pertain to a partially constrained subject-region model in which some of the coefficients are set equal to a complement-region weighted-average estimate. It is not necessary to re-estimate the weighted averages at each step; the regional fixed-effects model produces statistically consistent estimates of the coefficients and associated covariance matrices, regardless of any underlying cross-region constraints on the coefficients. The only complication in the procedure concerns the standard errors of the unconstrained coefficients in the constrained subject-region model, which depend on the uncertainty in the values of the constrained coefficients (Murphy and Topel, 1985) caused by uncertainty in the weighted averages (see the inverse term in the right-hand side of Equation A6). Using results from Murphy and Topel, it can be shown (see the derivation contained in the Supporting Information) that the covariance matrix for the vector of regional differentials for subject region *s* corresponding to the unconstrained coefficients of that region (denoted ) is consistently estimated by

(A8)

where  is the weighted nonlinear least squares (WNLS) estimate of the covariance matrix for the unrestricted coefficients, the constrained coefficients being set equal to the corresponding value of the weighted average of the complement-region coefficients, denoted here as ;  is the MSE of the region-*s* model prior to imposing any constraints;  is the MSE of the region-*s* model with some of the coefficients constrained (the model used to generate );  is a  identity matrix,  being the number of unconstrained coefficients in the region-*s* model;  is a  submatrix of the inverse of the covariance matrix of the region-*s* model prior to imposing any constraints, the  rows of which correspond to the unrestricted coefficients and the  columns correspond to the coefficients that have been constrained to equal the corresponding value of the weighted average of the complement-region coefficients; and  is the submatrix of the estimated covariance matrix for the weighted average of the complement-region coefficients for region *s* (i.e., the submatrix of the estimate of , see Equation (A6) above), the rows and columns of which pertain to the group of coefficients identified respectively by *A* and *B*, where *A* and *B* equal either *U*, for the unconstrained coefficients of the region-*s* model, or *C*, representing the constrained region-*s* coefficients. Note that the regional weights selected to minimize the covariance matrix  imply the quadratic form  is also minimized.

The collection of cross-region constraints, identified by the application of the stepwise procedure for each of *R*− 1 regions comprising a national model, can be applied toward the simultaneous estimation of the regional fixed-effects model, producing the hybrid model. Mathematically, the collection of constraints can be described as follows. Let  represent the vector of stacked regional differential vectors for each region, let  represent the vector of stacked regional fixed-effects coefficient values, and define **G** to be a *RK* × *RK* matrix that relates the stacked regional differentials to the regional fixed-effect coefficients, such that **δ** = **Gβ**. From the definition of **δ**_*s*_ given in Equation (A1), this implies

(A9)

where the **W**_*s*,*r*_ are defined in Equation (A3). Note that the restriction that the weights sum to one implies the rightmost column of block matrices in Equation (A9) equals minus one times the sum of the *R*− 1 leftmost columns, so **G** does not have full rank and is not invertible. A full rank formulation of the first *R*− 1 sets of regional differentials  can be obtained by modifying the **β** vector to consist of *R*− 1 sets of coefficient differences from the region *R* coefficient vector, so that , where  and  consists of the (*R*− 1) × (*R*− 1) upper-leftmost blocks of **G**,

(A10)

The *R*th set of regional differentials can also be expressed as a linear combination of , such that, , where . As  is nonsingular, we have , and the *R*th set of regional differentials are shown to be a linear combination of the other *R*− 1 sets of differentials. Thus, the constraints given by **δ**_*R*_ = 0 are not independent of other constraints applied to  and the stepwise procedure used to identify nonzero regional differentials need be applied to only *R*− 1 regions. As a result of the nonsingularity of , it is readily apparent that if all differentials are zero, so that , then  and all like-process coefficients across regions are equal, implying the hybrid model is equivalent to the national model.

Let **δ**^*C*^ refer to the collection of regional differentials determined by the stepwise procedure to be insignificantly different from zero and let **G**^*C*^ represent the rows of **G** corresponding to the  elements of **δ**^*C*^, so that **δ**^*C*^ = **G**^*C*^**β**. The failure to reject the hypothesis that , where  is a *K*^*C*^-element vector of zeros, leads to the relation

(A11)

a condition that implies *K*^*C*^ linear constraints across all the regional fixed-effects process coefficients. It is these constraints that are applied to the fixed-effects model to obtain the hybrid regionalized national model.

A consistent estimate of the covariance matrix for the constrained model takes the form (Gallant, 1987; see the Supporting Information for a derivation)

(A12)

where ,  being the partial derivative of the model estimate of load for monitored reach *i* (of a total of *N* monitored reaches that supply the dependent variable load data used to estimate the SPARROW model; see the description of the SPARROW model in the Supporting Information) with respect to the parameter vector, with derivatives evaluated at the constrained, WNLS estimates of the fixed-effects process coefficients, , and .  is positive definite (positive semidefinite if any of the coefficient estimates are constrained according to model constraints, such as that the source coefficients must be nonnegative), and Equation (A12) conforms with the linear constraints in Equation (A11) in that premultiplying  by **G**^*C*^ results in a zero matrix.

## SUPPORTING INFORMATION

**Data S1.** Description of the SPARROW methodology, a derivation of the covariance matrix for the case where some of the coefficients are set to predetermined values, a derivation of the covariance matrix for the case where coefficients are estimated under linear constraints, and tables containing the estimates and related statistics of the TN and TP national, regional fixed-effects, and hybrid models, are available as part of the online article.

Please note: Neither AWRA nor Wiley-Blackwell is responsible for the content or functionality of any supporting materials supplied by the authors. Any queries (other than missing material) should be directed to the corresponding author for the article.
