# Supplementary material for: The Regionalization of National-Scale SPARROW Models for Stream Nutrients
Source: J Am Water Resour Assoc. 2011 Oct;47(5):1151–72. doi: 10.1111/j.1752-1688.2011.00581.x (PMC3307635; doi:10.1111/j.1752-1688.2011.00581.x)
Supplement: Supplementary file 1 [file jawr0047-1151-SD1.pdf]

Supporting Information for: The Regionalization of National-Scale SPARROW Models for Stream Nutrients

Gregory E. Schwarz, Richard B. Alexander, Richard A. Smith, and Stephen D. Preston

Description of the SPARROW method

The SPARROW watershed modeling technique uses a hybrid statistical and process-based approach to estimate contaminant sources and contaminant transport in the surface waters of large-scale watersheds. A unique feature of the method concerns the spatial referencing of sources, delivery processes and monitoring stations in relation to a reach network, a referencing system that enables improved resolution of the effects sources and transport processes have on monitored loads. A mass balance accounting of load is achieved by linking all measured in-stream loads to identified upstream sources, and by requiring that the accumulation of load across sources and reaches be strictly additive. The dependent variable used in SPARROW is the long-term mean annual contaminant load, the analysis of which does not require an understanding of temporary storage, and thereby facilitates mass balance accounting of mean conditions in streams over long time periods. The spatial referencing noted above, in conjunction with mass balance accounting, allows the model to be used to assess the origin of in-stream contaminant load, both by source type and location, at any location along the reach network.

The SPARROW model specification includes three major structural components: contaminant sources, processes affecting the delivery of sources to streams in the reach network, and the aquatic processes affecting the transport of contaminants once they are in the stream network. A more detailed understanding of the SPARROW methodology can be gained by consideration of the basic equation that describes these elements. Let F_i^* be the model-estimated flux for contaminant leaving reach *i*. This flux is equal to the flux leaving adjacent reaches upstream of reach *i*, denoted F_j', where *j* indexes the set $J(i)$ of adjacent reaches upstream of reach *i*, plus additional flux that is generated within the incremental reach segment *i*. The set of adjacent upstream reaches will consist of two or more reaches if reach *i* is the result of a confluence, which is the most common condition, or one reach if reach *i* is a continuation of a single flowline, or no reaches if reach *i* is a headwater reach.

The functional relations determining reach *i* flux are given by

$$F_i^* = \left(\sum_{j \in J(i)} F_j' \right) \delta_i A\left(\mathbf{Z}_i^S, \mathbf{Z}_i^R; \boldsymbol{\theta}_S, \boldsymbol{\theta}_R \right) + \left(\sum_{m=1}^{M_S} S_{m,i} \alpha_m D_m \left(\mathbf{Z}_i^D; \boldsymbol{\theta}_D \right) \right) A'\left(\mathbf{Z}_i^S, \mathbf{Z}_i^R; \boldsymbol{\theta}_S, \boldsymbol{\theta}_R \right). \quad \text{(S1)}$$

The first summation term represents the amount of flux that leaves upstream reaches and is delivered downstream to reach *i*, where F_j' equals measured flux, F_j^M, if upstream reach *j* is monitored or, if it is not, is given by the model-estimated flux, F_j^*. δ_i is the fraction of

upstream flux delivered to reach *i*. This fraction is assigned by the reach network: if there are no diversions, then δ_i is set to 1; otherwise, the fraction is the fraction of streamflow diverted to reach *i*. $A(\cdot)$ is the aquatic delivery function representing attenuation processes acting on flux as it travels along the reach pathway. This function defines the fraction of flux entering reach *i* at the upstream node that is delivered to the reach's downstream node. The factor is a function of measured stream and reservoir characteristics, denoted by the vectors $\mathbf{Z}_i^S$ and $\mathbf{Z}_i^R$, with corresponding coefficient vectors $\boldsymbol{\theta}_S$ and $\boldsymbol{\theta}_R$. If reach *i* is a stream, then only the $\mathbf{Z}_i^S$ and $\boldsymbol{\theta}_S$ terms determine the value of $A(\cdot)$; conversely, if reach *i* is a reservoir then the terms that determine $A(\cdot)$ consist of $\mathbf{Z}_i^R$ and $\boldsymbol{\theta}_R$.

The second summation term in equation (S1) represents the amount of flux introduced to the stream network at reach *i*. This term is composed of the flux originating in specific sources, indexed by $m = 1,\ldots,M_S$. Associated with each source is a source variable, denoted S_m. Depending on the nature of the source, this variable could represent the mass of the source available for transport to streams, or it could be the area of a particular land use. The variable α_m is a source-specific coefficient (the collection of all source-specific coefficients is denoted subsequently by the vector $\boldsymbol{\alpha} = \{\alpha_1,\ldots,\alpha_{M_S}\}'$). This coefficient retains the units that convert source variable units to flux units. The function $D_m(\cdot)$ represents the land-to-water delivery factor. For sources associated with the landscape, this function along with the source-specific coefficient determines the amount of contaminant delivered to streams. The land-to-water delivery factor is a source-specific function of a vector of delivery variables, denoted by $\mathbf{Z}_i^D$, and an associated vector of coefficients $\boldsymbol{\theta}_D$. It is common practice to express the $\mathbf{Z}_i^D$ variables as differences from their respective mean for the modeled region, a transformation that expresses the source coefficient as a mean contaminant load delivered to the nearest stream segment in the reach network, per unit of the associated source variable. The last term in the equation, the function $A'(\cdot)$, represents the fraction of flux originating in and delivered to reach *i* that is transported to the reach's downstream node. This function is similar in form to the aquatic delivery factor defined in the first summation term of the flux equation; however, the default assumption in SPARROW models is that if reach *i* is classified as a stream, as opposed to a reservoir reach, the contaminants introduced to the reach from its incremental drainage area receive the square root of the reach's full in-stream delivery. This assumption is consistent with the notion that contaminants are introduced to the reach network at the midpoint of reach *i* and thus experience only half of the reach's time of travel. For reaches classified as reservoirs, the default assumption is that the contaminant receives the full attenuation associated with the reservoir.

The nonlinear model structure in equation (S1) contains several key features. The additive contaminant source components and multiplicative land and water transport terms are conceptually consistent with the physical mechanisms that explain the long-term mean rates of supply and movement of contaminants in watersheds. Total modeled flux for a reach is separated into its individual sources. Because this same decomposition is done

for all upstream reaches, it is possible in this framework to perform flux accounting, whereby total flux is attributed to its source components. All processes are spatially referenced with respect to the stream network according to the reach in which they operate. This means, for example, that a reservoir at reach *i* may affect the transport of contaminants entering the reach network upstream, but not downstream, of reach *i*. The additive source components also provide a mathematical structure in the model that preserves mass. This can be seen by noting that a doubling of each of the source variables $S_{m,i}$, along with a doubling of all upstream sources, as represented by a doubling of F_j', results in an exact doubling of modeled flux F_i^*. Finally, the modeled flux at any reach *i* is conditioned on monitored fluxes entering the stream network anywhere upstream of reach *i*. This approach to nested basins serves to isolate errors introduced in any upstream basin from incremental errors that arise in a downstream basin, making it defensible to treat nested basins as independent observations. Formally, the approach is similar to the way time series models condition predictions on past realizations of the series.

The relation between measured (F_i^M) and modeled (F_i^*) flux for monitored reach *i* is assumed to be given by

$$\ln F_i^M = \ln F_i^*(\boldsymbol{\beta}) + e_i, \qquad \text{(S2)}$$

where e_i is a random error that is independent across monitored basins , with mean zero and variance σ_i^2, and $F_i^*(\boldsymbol{\beta})$ is abbreviated notation for the explicit formula given in equation (S1), with $\boldsymbol{\beta} = \{\boldsymbol{\alpha}', \boldsymbol{\theta}_D', \boldsymbol{\theta}_S', \boldsymbol{\theta}_R'\}'$. Given a collection of *N* such observed basins, the method of weighted nonlinear least squares (WNLLS) is used to obtain estimates of the model coefficients, $\hat{\boldsymbol{\beta}}$, and their associated covariances. The weights in the WNLLS procedure are set to $w_i = N^{-1}\sum_{n=1}^{N}\sigma_n^2 \big/ \sigma_i^2$, a specification that preserves the average variance of the residuals. Typically, varying weights are used to account for differences in the accuracy of the mean load estimates obtained at monitoring stations. It is also common to estimate the model constraining the source coefficients and the land and aquatic delivery processes, $D_m(\cdot)$, $A(\cdot)$, and $A'(\cdot)$ to be non-negative, a restriction that insures predicted in-stream load is positive. Additional details on the specification and estimation of a SPARROW model are provided in Schwarz *et al.* (2006).

Derivation of the covariance matrix for the partially constrained regional model used in the stepwise procedure

The following describes the derivation of the covariance matrix for the unconstrained regional differentials, reported as equation (A8) in the Appendix to the article. Let *s* denote the subject region and define $R_{\neg s}$ as the sequence $r = 1, \ldots, s-1, s+1, \ldots, R$ indexing the set of $R-1$ complement regions. Define $\boldsymbol{\beta}_{\neg s} = \{\boldsymbol{\beta}'_{N_r\, r}, r \in R_{\neg s}\}'$ to be the vector of $(R-1)\times K$ region-specific

coefficient estimates for the complementary regions, and define $\mathbf{W}_s = \left[\mathbf{W}_{s,r}, r \in R_{\neg s}\right]$ to be the weighting matrix used to evaluate the weighted average of the complementary regional coefficients, $\mathbf{b}_s = \mathbf{W}_s \boldsymbol{\beta}_{\neg s}$. Define $\boldsymbol{\beta}_s^C$ to be the vector of K_s^C region-*s* coefficients that are constrained to equal the weighted average of the complement region coefficients, and let $\boldsymbol{\beta}_s^U$ and $\boldsymbol{\delta}_s^U$ represent the vector of $K_s^U = K - K_s^C$ unconstrained coefficients and associated regional differentials, where $\boldsymbol{\beta}_s^C = \mathbf{W}_s^C \boldsymbol{\beta}_{\neg s} = \mathbf{b}_s^C$ and $\boldsymbol{\beta}_s^U = \boldsymbol{\delta}_s^U + \mathbf{W}_s^U \boldsymbol{\beta}_{\neg s} = \boldsymbol{\delta}_s^U + \mathbf{b}_s^U$, with $\mathbf{W}_s^C$ and $\mathbf{b}_s^C$ representing the subset of rows of $\mathbf{W}_s$ and $\mathbf{b}_s$ corresponding to the constrained coefficients included in $\boldsymbol{\beta}_s^C$, and $\mathbf{W}_s^U$ and $\mathbf{b}_s^U$ representing the complement rows of $\mathbf{W}_s$ and $\mathbf{b}_s$ corresponding to the unrestricted coefficients included in $\boldsymbol{\beta}_s^U$.

The weighted nonlinear least squares objective function for region *s* is given by

$$Q_{N_s}\left(\boldsymbol{\beta}_s^C, \boldsymbol{\beta}_s^U; \mathbf{w}_s\right) = \left(\tilde{\mathbf{F}}_{N_s}^M - \tilde{\mathbf{F}}_{N_s}^*\left(\boldsymbol{\beta}_s^C, \boldsymbol{\beta}_s^U\right)\right)' \mathbf{D}_{N_s}\left(\mathbf{w}_s\right)\left(\tilde{\mathbf{F}}_{N_s}^M - \tilde{\mathbf{F}}_{N_s}^*\left(\boldsymbol{\beta}_s^C, \boldsymbol{\beta}_s^U\right)\right), \tag{S3}$$

where $\tilde{\mathbf{F}}_{N_s}^M$ is the vector corresponding to the N_s values of the natural logarithm of monitored load available in region *s*; $\tilde{\mathbf{F}}_{N_s}^*$ is the N_s- element vector of the natural logarithm of the modeled value of load in region *s*, assumed to have continuous first- and second-order derivatives with respect to all feasible values of its parameters, the coefficient vectors $\boldsymbol{\beta}_s^C$ and $\boldsymbol{\beta}_s^U$ (see equation (S3)); and $\mathbf{D}_{N_s}\left(\mathbf{w}_s\right)$ is a diagonal matrix with the vector $\mathbf{w}_s$, corresponding to the N_s observation-specific weights for region *s*, $w_{si} = N_s^{-1} \sum_{n=1}^{N_s} \sigma_{sn}^2 \Big/ \sigma_{si}^2$, arrayed along the diagonal. For simplicity, the observation-specific weights are assumed to be known; results presented in Amemiya (1985) imply that the covariance matrix derived below is unaffected if the weights are estimated consistently using a two-stage process whereby the squared values of the residuals estimated from a first-stage model, one with observation-specific weights set to 1, are regressed on various predictors, the predictions of which are used to compute the observation-specific weights in a second-stage model.

Let $\hat{\mathbf{b}}_s^C$ and $\hat{\mathbf{b}}_s^U$ represent the estimated values of $\mathbf{b}_s^C$ and $\mathbf{b}_s^U$, these estimates being based on the $N - N_s$ observations from the national model used to obtain the estimates $\hat{\mathbf{W}}_s$ and $\hat{\boldsymbol{\beta}}_{\neg s}$. An estimate of $\boldsymbol{\delta}_s^U$ can be obtained by substituting $\hat{\mathbf{b}}_s^C$ for $\boldsymbol{\beta}_s^C$, and $\boldsymbol{\delta}_s^U + \hat{\mathbf{b}}_s^U$ for $\boldsymbol{\beta}_s^U$ in (S3) and minimizing with respect $\boldsymbol{\delta}_s^U$. The K_s^U first-order conditions implied by this minimization are given by

$$Q_{N_s \boldsymbol{\beta}_s^{U'}}\left(\hat{\mathbf{b}}_s^C, \hat{\boldsymbol{\delta}}_s^U + \hat{\mathbf{b}}_s^U\right) \equiv \hat{Q}_{N_s \boldsymbol{\beta}_s^{U'}} = -2\left(\tilde{\mathbf{F}}_{N_s}^M - \hat{\tilde{\mathbf{F}}}_{N_s}^*\right)' \mathbf{D}_{N_s}\left(\mathbf{w}_s\right) \hat{\tilde{\mathbf{F}}}_{N_s \boldsymbol{\beta}_s^{U'}}^* = \mathbf{0}_{K_s^U}, \tag{S4}$$

where $Q_{N_s \boldsymbol{\beta}_s^{U\prime}}$ and $\hat{\tilde{\mathbf{F}}}^*_{N_s \boldsymbol{\beta}_s^{U\prime}}$ denote the partial derivatives of Q_{N_s} and $\tilde{\mathbf{F}}^*_{N_s}$ with respect to the row vector $\boldsymbol{\beta}_s^{U\prime}$, with $\tilde{\mathbf{F}}^*_{N_s}$ and $\tilde{\mathbf{F}}^*_{N_s \boldsymbol{\beta}_s^{U\prime}}$ evaluated at $\hat{\mathbf{b}}_s^C$ and $\hat{\boldsymbol{\delta}}_s^U + \hat{\mathbf{b}}_s^U$, and $\mathbf{0}_{K_s^U}$ is a K_s^U-element vector of zeros.

Let $\overline{\mathbf{b}}_s^C$, $\overline{\mathbf{b}}_s^U$, and $\overline{\boldsymbol{\delta}}_s^U$ denote the true values of the complementary region weighted average coefficients and the region-*s* unrestricted coefficient differentials. The first-order Taylor expansion of the first-order condition about these true values is given by

$$\hat{Q}_{N_s \boldsymbol{\beta}_s^{U\prime}} = 0 = \overline{Q}_{N_s \boldsymbol{\beta}_s^{U\prime}} + \left(\hat{\mathbf{b}}_s^C - \overline{\mathbf{b}}_s^C\right)' \widehat{Q}_{N_s \boldsymbol{\beta}_s^C \boldsymbol{\beta}_s^{U\prime}} + \left(\left(\hat{\boldsymbol{\delta}}_s^U - \overline{\boldsymbol{\delta}}_s^U\right)' + \left(\hat{\mathbf{b}}_s^U - \overline{\mathbf{b}}_s^U\right)'\right) \widehat{Q}_{N_s \boldsymbol{\beta}_s^U \boldsymbol{\beta}_s^{U\prime}}, \tag{S5}$$

where $\overline{Q}_{N_s \boldsymbol{\beta}_s^{U\prime}} = Q_{N_s \boldsymbol{\beta}_s^{U\prime}}\left(\overline{\mathbf{b}}_s^C, \overline{\boldsymbol{\delta}}_s^U + \overline{\mathbf{b}}_s^U\right)$, and $\widehat{Q}_{N_s \boldsymbol{\beta}_s^C \boldsymbol{\beta}_s^{U\prime}}$ and $\widehat{Q}_{N_s \boldsymbol{\beta}_s^U \boldsymbol{\beta}_s^{U\prime}}$ are evaluated at $\widehat{\mathbf{b}}_s = \eta \hat{\mathbf{b}}_s + (1-\eta)\overline{\mathbf{b}}_s$ and $\widehat{\boldsymbol{\delta}}_s^U = \eta \hat{\boldsymbol{\delta}}_s^U + (1-\eta)\overline{\boldsymbol{\delta}}_s^U$, for some value $\eta \in [0,1]$. Multiplication of (S5) by $\sqrt{N_s}$ and solving for $\left(\hat{\boldsymbol{\delta}}_s^U - \overline{\boldsymbol{\delta}}_s^U\right)$ gives

$$\begin{aligned}\sqrt{N_s}\left(\hat{\boldsymbol{\delta}}_s^U - \overline{\boldsymbol{\delta}}_s^U\right) = -\left(\frac{\widehat{Q}_{N_s \boldsymbol{\beta}_s^U \boldsymbol{\beta}_s^{U\prime}}}{N_s}\right)^{-1} \Bigg\{ \frac{\overline{Q}_{N_s \boldsymbol{\beta}_s^U}}{\sqrt{N_s}} + \sqrt{\frac{f_s}{1-f_s}} \Bigg[\left(\frac{\widehat{Q}_{N_s \boldsymbol{\beta}_s^U \boldsymbol{\beta}_s^{C\prime}}}{N_s}\right) \sqrt{N-N_s}\left(\hat{\mathbf{b}}_s^C - \overline{\mathbf{b}}_s^C\right) + \\ \left(\frac{\widehat{Q}_{N_s \boldsymbol{\beta}_s^U \boldsymbol{\beta}_s^{U\prime}}}{N_s}\right) \sqrt{N-N_s}\left(\hat{\mathbf{b}}_s^U - \overline{\mathbf{b}}_s^U\right) \Bigg] \Bigg\},\end{aligned} \tag{S6}$$

where $f_s = N_s / N$.

The first term in the braces of (S6) is

$$\begin{aligned}\frac{\overline{Q}_{N_s \boldsymbol{\beta}_s^U}}{\sqrt{N_s}} &= -N_s^{-\frac{1}{2}} 2\left(\tilde{\mathbf{F}}^*_{N_s \boldsymbol{\beta}_s^{U\prime}}\left(\overline{\mathbf{b}}_s^C, \overline{\boldsymbol{\delta}}_s^U + \overline{\mathbf{b}}_s^U\right)\right)' \mathbf{D}_{N_s}(\mathbf{w}_s)\left(\tilde{\mathbf{F}}^M_{N_s} - \tilde{\mathbf{F}}^*_{N_s}\left(\overline{\mathbf{b}}_s^C, \overline{\boldsymbol{\delta}}_s^U + \overline{\mathbf{b}}_s^U\right)\right) \\ &= -N_s^{-\frac{1}{2}} 2\left(\overline{\tilde{\mathbf{F}}}^*_{N_s \boldsymbol{\beta}_s^{U\prime}}\right)' \mathbf{D}_{N_s}(\mathbf{w}_s)\mathbf{e}_s,\end{aligned} \tag{S7}$$

where, by assumption, $\mathbf{e}_s = \tilde{\mathbf{F}}^M_{N_s} - \overline{\tilde{\mathbf{F}}}^*_{N_s}$ is an N_s-element vector of independent, normally distributed random variables having mean $\mathbf{0}_{N_s}$ and covariance $N_s^{-1}\sum_{i=1}^{N_s} \overline{\sigma}_i^2 \mathbf{D}^{-1}_{N_s}(\mathbf{w}_s)$.

The other terms in the brackets in (S6) contain the errors in the estimates of the complementary region coefficients, $\mathbf{b}_s^C$ and $\mathbf{b}_s^U$. Because these coefficients are estimated using data that exclude region *s*, these errors are independent of the other terms in (S6). The second-order partial derivative terms take the form

$$\frac{\widehat{Q}_{N_s\,\boldsymbol{\beta}_s^U\boldsymbol{\beta}_s^{C\prime}}}{N_s} = 2\frac{\left(\widehat{\widetilde{\mathbf{F}}}^*_{N_s\,\boldsymbol{\beta}_s^{U\prime}}\right)'\mathbf{D}_{N_s}\left(\mathbf{w}_s\right)\widehat{\widetilde{\mathbf{F}}}^*_{N_s\,\boldsymbol{\beta}_s^{C\prime}}}{N_s} - 2\frac{\sum_{i=1}^{N_s}\widehat{\widetilde{F}}^*_{i\,\boldsymbol{\beta}_s^U\boldsymbol{\beta}_s^{C\prime}}\widehat{w}_{s\,i}\left(e_{s\,i} + \overline{\widetilde{F}}_i - \widehat{\widetilde{F}}_i^*\right)}{N_s} \tag{S8}$$

and

$$\frac{\widehat{Q}_{N_s\,\boldsymbol{\beta}_s^U\boldsymbol{\beta}_s^{U\prime}}}{N_s} = 2\frac{\left(\widehat{\widetilde{\mathbf{F}}}^*_{N_s\,\boldsymbol{\beta}_s^{U\prime}}\right)'\mathbf{D}_{N_s}\left(\mathbf{w}_s\right)\widehat{\widetilde{\mathbf{F}}}^*_{N_s\,\boldsymbol{\beta}_s^{U\prime}}}{N_s} - 2\frac{\sum_{i=1}^{N_s}\widehat{\widetilde{F}}^*_{i\,\boldsymbol{\beta}_s^U\boldsymbol{\beta}_s^{U\prime}}\widehat{w}_{s\,i}\left(e_{s\,i} + \overline{\widetilde{F}}_i - \widehat{\widetilde{F}}_i^*\right)}{N_s}, \tag{S9}$$

where $e_{s\,i}$, $\overline{\widetilde{F}}_i$ and $\widehat{\widetilde{F}}_i^*$ are individual elements of $\mathbf{e}_s$, $\overline{\widetilde{\mathbf{F}}}_{N_s}$ and $\widehat{\widetilde{\mathbf{F}}}^*_{N_s}$.

Amemiya (1985, Theorem 4.3.2) describes general conditions that insure the consistent estimation of the parameters and associated asymptotic covariance matrices of a nonlinear model. Assuming those conditions hold for both the region *s* model and for the independent models pertaining to the regions complementary to *s*, as sample sizes in all regions go to infinity we obtain consistent estimates of $\boldsymbol{\beta}_{\neg s}$ and $\mathbf{W}_s$, implying $\hat{\mathbf{b}}_s$ is consistent, as is the estimate $\hat{\boldsymbol{\delta}}_s^U$, thereby guaranteeing that $\operatorname{plim}\hat{\mathbf{b}}_s = \overline{\mathbf{b}}_s$ and $\operatorname{plim}\hat{\boldsymbol{\delta}}_s^U = \overline{\boldsymbol{\delta}}_s^U$. Additionally, the conditions for Amemiya's Theorem 4.3.2, along with the statistical independence of the regions, imply that $\sqrt{N-N_s}\left(\hat{\mathbf{b}}_s - \overline{\mathbf{b}}_s\right)$ has an asymptotic distribution that is multivariate normal with a zero mean vector and covariance matrix equal to

$$\tilde{\mathbf{V}}\left(\hat{\mathbf{b}}_s\right) = \left(\sum\nolimits_{j\neq s} s_j\tilde{\mathbf{V}}_j^{-1}\right)^{-1}, \tag{S10}$$

where $s_j = \lim_{N,N_s,N_j\to\infty} N_j/\left(N-N_s\right)$ and $\tilde{\mathbf{V}}_j$ is the asymptotic covariance matrix of $\sqrt{N_j}\left(\hat{\boldsymbol{\beta}}_j - \overline{\boldsymbol{\beta}}_j\right)$. Note that because $\tilde{\mathbf{V}}_j = \operatorname{plim} N_j\mathbf{V}_j$, where $\mathbf{V}_j$ is the estimated covariance matrix for region *j* used in Proposition 1 of the Appendix, we have $\left(\sum\nolimits_{j\neq s} s_j\tilde{\mathbf{V}}_j^{-1}\right)^{-1} = \operatorname{plim}\left(N-N_s\right)\hat{\mathbf{V}}\left(\hat{\mathbf{b}}_s\right)$, where $\hat{\mathbf{V}}\left(\hat{\mathbf{b}}_s\right) = \left(\sum\nolimits_{j\neq s}\mathbf{V}_j^{-1}\right)^{-1}$, which links the asymptotic covariance matrix derived here to the covariance matrix for the weighted average of the region-specific coefficient vectors reported in the article.

The conditions for Amemiya's Theorem 4.3.2 further imply that $N_s^{-1/2}\overline{Q}_{N_s\,\boldsymbol{\beta}_s^U}$ is distributed multivariate normal with a zero mean vector and nonsingular asymptotic covariance matrix

$$\lim_{N_s\to\infty}\left(\frac{\sum_{i=1}^{N_s}\overline{\sigma}_i^2}{N_s}\right)\frac{4\left(\widetilde{\overline{\mathbf{F}}}^*_{N_s\boldsymbol{\beta}_s^{U'}}\right)'\mathbf{D}_{N_s}(\mathbf{w}_s)\widetilde{\overline{\mathbf{F}}}^*_{N_s\boldsymbol{\beta}_s^{U'}}}{N_s}, \tag{S11}$$

and the probability limits of the sample size-normalized second-order derivatives of the objective function are

$$\operatorname{plim}\frac{\widehat{Q}_{N_s\boldsymbol{\beta}_s^U\boldsymbol{\beta}_s^{C'}}}{N_s}=\lim_{N_s\to\infty}\frac{2\left(\widetilde{\overline{\mathbf{F}}}^*_{N_s\boldsymbol{\beta}_s^{U'}}\right)'\mathbf{D}_{N_s}(\mathbf{w}_s)\widetilde{\overline{\mathbf{F}}}^*_{N_s\boldsymbol{\beta}_s^{C'}}}{N_s}\equiv 2\mathbf{A}_s \tag{S12}$$

and

$$\operatorname{plim}\frac{\widehat{Q}_{N_s\boldsymbol{\beta}_s^U\boldsymbol{\beta}_s^{U'}}}{N_s}=\lim_{N_s\to\infty}\frac{2\left(\widetilde{\overline{\mathbf{F}}}^*_{N_s\boldsymbol{\beta}_s^{U'}}\right)'\mathbf{D}_{N_s}(\mathbf{w}_s)\widetilde{\overline{\mathbf{F}}}^*_{N_s\boldsymbol{\beta}_s^{U'}}}{N_s}\equiv 2\mathbf{B}_s\,. \tag{S13}$$

Given these results, and given the statistical independence between $N_s^{-1/2}\overline{Q}_{N_s\boldsymbol{\beta}_s^U}$ and $\hat{\mathbf{b}}_s$, expression (S6) implies $\sqrt{N_s}\left(\hat{\boldsymbol{\delta}}_s^U-\overline{\boldsymbol{\delta}}_s^U\right)$ has an asymptotic distribution that is multivariate normal with zero mean vector and asymptotic covariance matrix given by

$$\widetilde{\mathbf{V}}\left(\hat{\boldsymbol{\delta}}_s^U\right)=\widetilde{\sigma}_s^2\mathbf{B}_s^{-1}+\frac{\widetilde{f}_s}{1-\widetilde{f}_s}\left[\mathbf{B}_s^{-1}\mathbf{A}_s\quad \mathbf{I}_{K^U}\right]\begin{bmatrix}\widetilde{\mathbf{V}}\left(\hat{\mathbf{b}}_s\right)_{[C,C]} & \widetilde{\mathbf{V}}\left(\hat{\mathbf{b}}_s\right)_{[U,C]}\\ \widetilde{\mathbf{V}}\left(\hat{\mathbf{b}}_s\right)_{[C,U]} & \widetilde{\mathbf{V}}\left(\hat{\mathbf{b}}_s\right)_{[U,U]}\end{bmatrix}\begin{bmatrix}\mathbf{A}_s'\mathbf{B}_s^{-1}\\ \mathbf{I}_{K^U}\end{bmatrix}, \tag{S14}$$

where $\widetilde{\sigma}_s^2=\lim_{N_s\to\infty}N_s^{-1}\sum_{i=1}^{N_s}\overline{\sigma}_i^2$, $\widetilde{f}_s=\lim_{N_s,N\to\infty}\frac{N_s}{N}$, and $\widetilde{\mathbf{V}}\left(\hat{\mathbf{b}}_s\right)_{[X,Y]}$ is the sub-matrix of the asymptotic covariance matrix $\widetilde{\mathbf{V}}\left(\hat{\mathbf{b}}_s\right)$ having rows corresponding to elements of vector $\hat{\mathbf{b}}_s^X$ and columns corresponding to the elements of vector $\hat{\mathbf{b}}_s^Y$. Note that except for the term involving $\widetilde{f}_s$, (S14) has the same form as the asymptotic variance derived by Murphy and Topel (1985). Murphy and Topel made the implicit assumption that in the limit the absolute difference between the number of observations used to estimate the prior model (here, the model used to estimate $\hat{\mathbf{b}}_s$) and the number of observations used to estimate the subsequent constrained model (here, the model used to estimate $\hat{\boldsymbol{\delta}}_s^U$) is bounded. In that case $\left|(N-N_s)-N_s\right|=\left|N-2N_s\right|\le M<\infty$, where M is a fixed upper bound on the difference in observations, and $\lim_{N,N_s\to\infty}\left|(N-2N_s)/N\right|=\left|1-2\lim_{N,N_s\to\infty}(N_s/N)\right|\le\lim_{N,N_s\to\infty}(M/N)=0$, implying $\widetilde{f}_s=1/2$ and the term

involving $\tilde{f}_s$ in (S14) gets dropped from the expression. In the present analysis we allow for the possibility that sampling across regions is proportional, in which case the absolute difference between the number of observations in the various regions is not bounded, leading to the retention of the $\tilde{f}_s$ term in (S14). Ultimately, the issue is moot because the sample-estimated variances are not normalized by the number of observations, in which case the $\tilde{f}_s$ term disappears from the variance expression (see below).

A consistent estimate of $\tilde{\mathbf{V}}(\hat{\boldsymbol{\delta}}_s^U)$ can be obtained as follows. First, obtain weighted nonlinear least squares estimates of the region-specific process coefficients for the complement regions. These estimates can be obtained by minimizing an objective function such as (S3) for each regional model independently, without cross-region constraints on the coefficients, to obtain consistent estimates of the region-specific coefficient vectors, $\hat{\boldsymbol{\beta}}_{N_s r}$, and associated covariance matrix, $\hat{\mathbf{V}}_r$, for $r = 1, \ldots, R$. Stack the $r \in R_{\neg s}$ region-specific coefficient vectors to form $\hat{\boldsymbol{\beta}}_{\neg s}$, use equation (A3) and the $r \in R_{\neg s}$ covariance matrices to obtain a consistent estimate of the weight matrix, $\hat{\mathbf{W}}_s$, and estimate the weighted average of the complement region coefficient vector as $\hat{\mathbf{b}}_s = \hat{\mathbf{W}}_s \hat{\boldsymbol{\beta}}_{\neg s}$. An estimate of the associated covariance matrix is $\hat{\mathbf{V}}(\hat{\mathbf{b}}_s) = \left(\sum_{r \neq s} \hat{\mathbf{V}}_r^{-1}\right)^{-1}$, with $(N - N_s)\hat{\mathbf{V}}(\hat{\mathbf{b}}_s)$ being a consistent estimate of $\tilde{\mathbf{V}}(\hat{\mathbf{b}}_s)$.

Next, substitute the subset $\hat{\mathbf{b}}_s^C$ of $\hat{\mathbf{b}}_s$ for $\boldsymbol{\beta}_s^C$ in (S3) and minimize with respect to $\boldsymbol{\beta}_s^U$ to obtain estimates of the region-*s* unconstrained coefficients, $\hat{\boldsymbol{\beta}}_s^U$, and associated covariance matrix $\hat{\mathbf{V}}_s^U$, with $N_s \hat{\mathbf{V}}_s^U$ being a consistent estimate of $\tilde{\sigma}_s^2 \mathbf{B}_s^{-1}$. Let $\hat{\sigma}_s^{2U}$ represent the mean squared error (MSE) formed from the estimated weighted residuals, $\mathbf{D}_{N_s}^{1/2}(\mathbf{w}_s)\hat{\mathbf{e}}_s$, from the model used to estimate $\hat{\boldsymbol{\beta}}_s^U$, and let $\hat{\sigma}_s^2$ be the MSE formed from the estimated weighted residuals for the model used to obtain the unconstrained coefficient estimates, $\hat{\boldsymbol{\beta}}_s$, for region *s*. A consistent estimate of $\mathbf{B}_s^{-1}\mathbf{A}_s$ is $\left(\hat{\sigma}_s^2 / \hat{\sigma}_s^{2U}\right)\hat{\mathbf{V}}_s^U \hat{\mathbf{V}}_s^{-1}{}_{[U,C]}$, where $\hat{\mathbf{V}}_s^{-1}{}_{[U,C]}$ represents a sub-matrix of $\hat{\mathbf{V}}_s^{-1}$ having rows corresponding to the elements of the vector of unconstrained coefficients, $\boldsymbol{\beta}_s^U$, and columns corresponding to the elements of the vector of constrained coefficients, $\boldsymbol{\beta}_s^C$. Therefore, the sample covariance matrix used to test the statistical significance of the estimate of the region-*s* unconstrained differential vector $\hat{\boldsymbol{\delta}}_s^U = \hat{\boldsymbol{\beta}}_s^U - \hat{\mathbf{b}}_s^U$ is given by

$$\hat{\mathbf{V}}(\hat{\boldsymbol{\delta}}_s^U) = \hat{\mathbf{V}}_s^U + \left[\left(\hat{\sigma}_s^2/\hat{\sigma}_s^{2U}\right)\hat{\mathbf{V}}_s^U \hat{\mathbf{V}}_s^{-1}{}_{[U,C]} \quad \mathbf{I}_{K^U}\right] \begin{bmatrix} \hat{\mathbf{V}}(\hat{\mathbf{b}}_s)_{[C,C]} & \hat{\mathbf{V}}(\hat{\mathbf{b}}_s)_{[C,U]} \\ \hat{\mathbf{V}}(\hat{\mathbf{b}}_s)_{[U,C]} & \hat{\mathbf{V}}(\hat{\mathbf{b}}_s)_{[U,U]} \end{bmatrix} \begin{bmatrix} \left(\hat{\sigma}_s^2/\hat{\sigma}_s^{2U}\right)\left(\hat{\mathbf{V}}_s^{-1}{}_{[U,C]}\right)' \hat{\mathbf{V}}_s^U \\ \mathbf{I}_{K^U} \end{bmatrix}, \quad \text{(S15)}$$

with $N_s \hat{\mathbf{V}}(\hat{\mathbf{b}}_s)$ being a consistent estimator of $\tilde{\mathbf{V}}(\hat{\mathbf{b}}_s)$ given in (S14), thus establishing the validity of equation (A8) of the Appendix of the article.

Derivation of the covariance matrix for the constrained estimation of the regionalized, fixed-effects national model

The following describes the derivation of the covariance matrix for the constrained estimation of the regional fixed-effects model (i.e., the hybrid model), appearing in the article as equation (A12). This derivation follows the method described by Gallant (1987).
The Lagrangian representing the constrained form of the weighted nonlinear least squares objective function is given by

$$L = Q_N^*(\boldsymbol{\beta};\mathbf{w}) + \boldsymbol{\lambda}'\mathbf{G}^C\boldsymbol{\beta}, \tag{S16}$$

with the weighted sum of squares objective given by

$$Q_N^*(\boldsymbol{\beta};\mathbf{w}_s) = \left(\tilde{\mathbf{F}}_N^M - \tilde{\mathbf{F}}_N^*(\boldsymbol{\beta})\right)'\mathbf{D}_N(\mathbf{w})\left(\tilde{\mathbf{F}}_N^M - \tilde{\mathbf{F}}_N^*(\boldsymbol{\beta})\right), \tag{S17}$$

where $\tilde{\mathbf{F}}_N^M$ is the vector corresponding to the N values of the natural logarithm of monitored load available for the entire nation; $\tilde{\mathbf{F}}_N^*$ is the N - element vector of the natural logarithm of the modeled value of load, assumed to have continuous first- and second-order derivatives with respect to all feasible values of its parameters, the regional fixed-effect process coefficient vector $\boldsymbol{\beta}$; $\mathbf{D}_N(\mathbf{w})$ is a diagonal matrix with the vector $\mathbf{w}$, corresponding to the N observation-specific weights, $w_i = \bar{\sigma}^2/\sigma_i^2$, $\bar{\sigma}^2 = N^{-1}\sum_{n=1}^{N}\sigma_n^2$, arrayed along the diagonal; $\mathbf{G}^C$ is the $K^C \times RK$ matrix of linear constraint coefficients described in the main text, derived from **G** given in equation (A9); and $\boldsymbol{\lambda}$ is the $K^C \times 1$ vector of Lagrangian constraint parameters. To simplify the analysis, the observation-specific weight vector, **w**, is assumed to be known.

The first-order conditions for minimization of (S16) with respect to $\boldsymbol{\beta}$ and $\boldsymbol{\lambda}$, to obtain the estimates $\hat{\boldsymbol{\beta}}$ and $\hat{\boldsymbol{\lambda}}$, are given by (assuming all constraints are binding)

$$L_{\boldsymbol{\beta}} = Q_{N\boldsymbol{\beta}}^*(\hat{\boldsymbol{\beta}};\mathbf{w}) + \mathbf{G}^{C\prime}\hat{\boldsymbol{\lambda}} = \mathbf{0}_K, \; L_{\boldsymbol{\lambda}} = \mathbf{G}^C\hat{\boldsymbol{\beta}} = \mathbf{0}_{K^C}, \tag{S18}$$

where $\mathbf{0}_n$ is an $n \times 1$ vector of zeros.

The first-order Taylor expansion of $Q_{N\boldsymbol{\beta}}^*(\hat{\boldsymbol{\beta}};\mathbf{w})$ about the true value of the coefficients, $\bar{\boldsymbol{\beta}}$, is given by

$$Q_{N\boldsymbol{\beta}}^*(\hat{\boldsymbol{\beta}};\mathbf{w}) = Q_{N\boldsymbol{\beta}}^*(\bar{\boldsymbol{\beta}};\mathbf{w}) + Q_{N\boldsymbol{\beta}\boldsymbol{\beta}'}^*(\breve{\boldsymbol{\beta}};\mathbf{w})(\hat{\boldsymbol{\beta}} - \bar{\boldsymbol{\beta}}), \tag{S19}$$

where $\breve{\boldsymbol{\beta}} = \eta\hat{\boldsymbol{\beta}} + (1-\eta)\bar{\boldsymbol{\beta}}$, for some value $\eta \in [0,1]$.

Substitution of the right-hand side of (S19) for $Q^{*}_{N\boldsymbol{\beta}}\left(\hat{\boldsymbol{\beta}};\mathbf{w}\right)$ in the first equation of (S18) and, assuming $Q^{*}_{N\boldsymbol{\beta\beta}'}\left(\hat{\boldsymbol{\beta}};\mathbf{w}\right)$ is nonsingular, solving for $\hat{\boldsymbol{\beta}}-\overline{\boldsymbol{\beta}}$, yields

$$\hat{\boldsymbol{\beta}}-\overline{\boldsymbol{\beta}}=-\left(Q^{*}_{N\boldsymbol{\beta\beta}'}\left(\hat{\boldsymbol{\beta}};\mathbf{w}\right)\right)^{-1}\left(Q^{*}_{N\boldsymbol{\beta}}\left(\overline{\boldsymbol{\beta}};\mathbf{w}\right)+\mathbf{G}^{C\prime}\hat{\boldsymbol{\lambda}}\right). \tag{S20}$$

Because the constraints must be satisfied for both $\hat{\boldsymbol{\beta}}$ and $\overline{\boldsymbol{\beta}}$, pre-multiplication of (S20) by $\mathbf{G}^{C}$ gives

$$\mathbf{G}^{C}\left(\hat{\boldsymbol{\beta}}-\overline{\boldsymbol{\beta}}\right)=\mathbf{0}_{K^{C}}=-\mathbf{G}^{C}\left(Q^{*}_{N\boldsymbol{\beta\beta}'}\left(\hat{\boldsymbol{\beta}};\mathbf{w}\right)\right)^{-1}\left(Q^{*}_{N\boldsymbol{\beta}}\left(\overline{\boldsymbol{\beta}};\mathbf{w}\right)+\mathbf{G}^{C\prime}\hat{\boldsymbol{\lambda}}\right), \tag{S21}$$

which, assuming $\mathbf{G}^{C}\left(Q^{*}_{N\boldsymbol{\beta\beta}'}\left(\hat{\boldsymbol{\beta}};\mathbf{w}\right)\right)^{-1}\mathbf{G}^{C\prime}$ is a nonsingular matrix, provides the solution for the Lagrangian constraint parameters

$$\hat{\boldsymbol{\lambda}}=-\left(\mathbf{G}^{C}\left(Q^{*}_{N\boldsymbol{\beta\beta}'}\left(\hat{\boldsymbol{\beta}};\mathbf{w}\right)\right)^{-1}\mathbf{G}^{C\prime}\right)^{-1}\mathbf{G}^{C}\left(Q^{*}_{N\boldsymbol{\beta\beta}'}\left(\hat{\boldsymbol{\beta}};\mathbf{w}\right)\right)^{-1}Q^{*}_{N\boldsymbol{\beta}}\left(\overline{\boldsymbol{\beta}};\mathbf{w}\right). \tag{S22}$$

Substitution of this result into (S20), and multiplication through by $\sqrt{N}$, gives

$$\begin{aligned}\sqrt{N}\left(\hat{\boldsymbol{\beta}}-\overline{\boldsymbol{\beta}}\right)=&\left(\left(\frac{Q^{*}_{N\boldsymbol{\beta\beta}'}\left(\hat{\boldsymbol{\beta}};\mathbf{w}\right)}{N}\right)^{-1}\mathbf{G}^{C\prime}\left(\mathbf{G}^{C}\left(\frac{Q^{*}_{N\boldsymbol{\beta\beta}'}\left(\hat{\boldsymbol{\beta}};\mathbf{w}\right)}{N}\right)^{-1}\mathbf{G}^{C\prime}\right)^{-1}\mathbf{G}^{C}-\mathbf{I}_{RK}\right)\times\\&\left(\frac{Q^{*}_{N\boldsymbol{\beta\beta}'}\left(\hat{\boldsymbol{\beta}};\mathbf{w}\right)}{N}\right)^{-1}\frac{Q^{*}_{N\boldsymbol{\beta}}\left(\overline{\boldsymbol{\beta}};\mathbf{w}\right)}{\sqrt{N}},\end{aligned} \tag{S23}$$

where $\mathbf{I}_{RK}$ is an $RK\times RK$ identity matrix.

The vector $N^{-\frac{1}{2}}Q^{*}_{N\boldsymbol{\beta}}\left(\overline{\boldsymbol{\beta}};\mathbf{w}\right)$ can be expressed in terms of the model functions and variables as

$$\frac{Q^{*}_{N\boldsymbol{\beta}}\left(\overline{\boldsymbol{\beta}};\mathbf{w}\right)}{\sqrt{N}}=-N^{-\frac{1}{2}}2\left(\tilde{\mathbf{F}}^{*}_{N\boldsymbol{\beta}'}\left(\overline{\boldsymbol{\beta}}\right)\right)'\mathbf{D}_{N}\left(\mathbf{w}\right)\left(\tilde{\mathbf{F}}^{M}_{N}-\tilde{\mathbf{F}}^{*}_{N}\left(\overline{\boldsymbol{\beta}}\right)\right)=-N^{-\frac{1}{2}}2\left(\tilde{\mathbf{F}}^{*}_{N\boldsymbol{\beta}'}\left(\overline{\boldsymbol{\beta}}\right)\right)'\mathbf{D}_{N}\left(\mathbf{w}\right)\mathbf{e}, \tag{S24}$$

where, by assumption, $\mathbf{e}$ is an *N*-element vector of independent, normally distributed random variables having mean $\mathbf{0}_{N}$ and assumed covariance matrix $N^{-1}\left(\sum_{i=1}^{N}\overline{\sigma}_{i}^{2}\right)\mathbf{D}_{N}^{-1}\left(\mathbf{w}\right)$. Therefore, $N^{-\frac{1}{2}}Q^{*}_{N\boldsymbol{\beta}}\left(\overline{\boldsymbol{\beta}};\mathbf{w}\right)$ is distributed multivariate normal with mean $\mathbf{0}_{RK}$ and covariance matrix

$$E\left[\frac{Q^*_{N\boldsymbol{\beta}}\left(\overline{\boldsymbol{\beta}};\mathbf{w}\right)Q^*_{N\boldsymbol{\beta}}\left(\overline{\boldsymbol{\beta}};\mathbf{w}\right)'}{N}\right]=4\overline{\sigma}^2\frac{\sum_{i=1}^{N}w_i\widetilde{F}^*_{i\boldsymbol{\beta}}\left(\overline{\boldsymbol{\beta}}\right)\widetilde{F}^*_{i\boldsymbol{\beta}'}\left(\overline{\boldsymbol{\beta}}\right)}{N},\tag{S25}$$

the asymptotic limit of which is given by

$$\lim_{N\to\infty}E\left[\frac{Q^*_{N\boldsymbol{\beta}}\left(\overline{\boldsymbol{\beta}};\mathbf{w}\right)Q^*_{N\boldsymbol{\beta}}\left(\overline{\boldsymbol{\beta}};\mathbf{w}\right)'}{N}\right]=4\widetilde{\sigma}^2\mathbf{A},\tag{S26}$$

where $\widetilde{\sigma}^2=\lim_{N\to\infty}N^{-1}\sum_{i=1}^{N}\overline{\sigma}_i^2$, and $\mathbf{A}=\lim_{N\to\infty}N^{-1}\sum_{i=1}^{N}w_i\widetilde{F}^*_{i\boldsymbol{\beta}}\left(\overline{\boldsymbol{\beta}}\right)\widetilde{F}^*_{i\boldsymbol{\beta}'}\left(\overline{\boldsymbol{\beta}}\right)$.

Under the conditions stated for Theorem 4.3.2 in Amemiya (1985), the probability limit of $\hat{\boldsymbol{\beta}}$ is $\overline{\boldsymbol{\beta}}$. Because $\widehat{\boldsymbol{\beta}}$ lies between $\hat{\boldsymbol{\beta}}$ and $\overline{\boldsymbol{\beta}}$, it must also have a probability limit of $\overline{\boldsymbol{\beta}}$. Moreover, the conditions imply the probability limit of $N^{-1}Q^*_{N\boldsymbol{\beta}\boldsymbol{\beta}'}\left(\widehat{\boldsymbol{\beta}};\mathbf{w}\right)$ is given by

$$\operatorname{plim}\frac{Q^*_{N\boldsymbol{\beta}\boldsymbol{\beta}'}\left(\widehat{\boldsymbol{\beta}};\mathbf{w}\right)}{N}=\lim_{N\to\infty}\frac{2\sum_{i=1}^{N}w_i\widetilde{F}^*_{i\boldsymbol{\beta}}\left(\overline{\boldsymbol{\beta}}\right)\widetilde{F}^*_{i\boldsymbol{\beta}'}\left(\overline{\boldsymbol{\beta}}\right)}{N}\equiv 2\mathbf{A}.\tag{S27}$$

Thus, assuming the conditions for Amemiya’s Theorem 4.3.2 hold, the asymptotic distribution of $\sqrt{N}\left(\hat{\boldsymbol{\beta}}-\overline{\boldsymbol{\beta}}\right)$ is multivariate normal with mean $\mathbf{0}_{RK}$ and asymptotic covariance matrix given by

$$\begin{aligned}\widetilde{\mathbf{V}}\left(\hat{\boldsymbol{\beta}}\right)&=\widetilde{\sigma}^2\left(\mathbf{A}^{-1}\mathbf{G}^{C\prime}\left(\mathbf{G}^C\mathbf{A}^{-1}\mathbf{G}^{C\prime}\right)^{-1}\mathbf{G}^C-\mathbf{I}_{RK}\right)\mathbf{A}^{-1}\left(\mathbf{A}^{-1}\mathbf{G}^{C\prime}\left(\mathbf{G}^C\mathbf{A}^{-1}\mathbf{G}^{C\prime}\right)^{-1}\mathbf{G}^C-\mathbf{I}_{RK}\right)',\\&=\widetilde{\sigma}^2\left(\mathbf{A}^{-1}-\mathbf{A}^{-1}\mathbf{G}^{C\prime}\left(\mathbf{G}^C\mathbf{A}^{-1}\mathbf{G}^{C\prime}\right)^{-1}\mathbf{G}^C\mathbf{A}^{-1}\right).\end{aligned}\tag{S28}$$

Define $\mathbf{V}_0$ to be the sample estimate $\mathbf{V}_0=\overline{\sigma}^2\left(\sum_{i=1}^{N}w_i\widetilde{F}^*_{i\boldsymbol{\beta}}\left(\hat{\boldsymbol{\beta}}\right)\widetilde{F}^*_{i\boldsymbol{\beta}'}\left(\hat{\boldsymbol{\beta}}\right)\right)^{-1}$, and define the covariance matrix sample estimate to be

$$\mathbf{V}\left(\hat{\boldsymbol{\beta}}\right)=\mathbf{V}_0-\mathbf{V}_0\mathbf{G}^{C\prime}\left(\mathbf{G}^C\mathbf{V}_0\mathbf{G}^{C\prime}\right)^{-1}\mathbf{G}^C\mathbf{V}_0.\tag{S29}$$

Under the conditions of Theorem 4.3.2, $\operatorname{plim}N\mathbf{V}_0=\widetilde{\sigma}^2\mathbf{A}^{-1}$, implying $N\mathbf{V}\left(\hat{\boldsymbol{\beta}}\right)$ is a consistent estimate of $\widetilde{\mathbf{V}}\left(\hat{\boldsymbol{\beta}}\right)$ given in (S28), thus establishing the validity of equation (A12) in the article.

Complete results for the hybrid regionalized national model, the regionalized fixed-effects model, the weighted averages of the fixed-effects estimates, and the national model

The following tables present the complete estimation results, for each of the three regions, for both total nitrogen (TN, Table S1) and total phosphorus (TP, Table S2). Each table reports the coefficient estimates of the national model (as reported in Alexander *et al*., 2008); the regionalized fixed-effects model; and the hybrid model. Also reported are the standard errors and p-values for each coefficient estimate, an indicator of which process coefficients are subject to a physical bound on feasible values for the coefficient (the bound being that the coefficient must be greater than or equal to 0; applied to all source and aquatic removal coefficients), indicators of which hybrid and regional model estimates for which the physical bound is binding (that is, the estimate is forced to zero in order to meet a physical bound that the value be non-negative), and an indicator of which hybrid estimates have a binding cross-region constraint (that is, which hybrid coefficients are constrained to equal a weighted average of the complement regions' hybrid model estimates.

The standard errors for the hybrid model estimates are based on the covariance matrix computed using equation (A12) of the Appendix for the article. The standard errors for the regionalized fixed-effects estimates are based on covariance matrices computed using standard nonlinear methods and derived from the results of the regional fixed-effects model (referred to in the article as the $\mathbf{V}_r$ matrices; see, for example, equation (A2) in the Appendix). Note that because model residuals for a SPARROW model are assumed to be independent, and because all regions are delineated at a monitoring station, the covariances corresponding to fixed-effect coefficients from different regions are exactly zero. Standard errors for the regionalized fixed-effects and hybrid models are unreported if the corresponding coefficient is constrained in estimation to equal the physical bound of zero (source and aquatic removal rate coefficients are constrained to be non-negative; the coefficient is set to zero if the constraint is binding). The standard errors for the national model are taken from Alexander *et al*. (2008). The reported p-values are based on a Student's t distribution, using errors degrees of freedom as reported in Table 2. Reported p-values are one-tailed for coefficients subject to a model constraint (coefficients with a "1" in the column titled "Coefficient subject to model constraint"); otherwise, a two-tailed p-value is reported.

TABLE S1. Coefficient estimates and associated statistics for the total nitrogen (TN) models. See the above discussion for a description of each field presented in the table. Region abbreviations are E for East, NW for Northwest, and SW for Southwest (see Figure 1).

Parameter	Region	Coefficient estimates			Standard errors[a]			p-values[a]			If coefficient subject to a physical constraint[b]	If regional fixed-effect model estimate physically constrained[c]	If hybrid model estimate physically constrained[d]	If hybrid model estimate cross-region constrained[e]
		National model	Regional fixed-effects model	Hybrid model	National model	Regional fixed-effects model	Hybrid model	National model	Regional fixed-effects model	Hybrid model				
Urban	E	2.575	1.956	2.378	0.322	0.314	0.375	<0.001	<0.001	<0.001	1	0	0	0
Atmospheric deposition	E	0.694	0.878	0.599	0.144	0.206	0.183	<0.001	<0.001	<0.001	1	0	0	0
Corn/soybean	E	0.164	0.108	0.142	0.024	0.026	0.032	<0.001	<0.001	<0.001	1	0	0	0
Alfalfa	E	0.082	0.018	0.059	0.058	0.063	0.050	0.076	0.388	0.117	1	0	0	0
Wheat	E	0.137	0.000	0.000	0.060			0.011			1	1	1	0
Other crops	E	0.071	0.007	0.052	0.030	0.023	0.024	0.010	0.387	0.015	1	0	0	0
Pasture/range	E	0.063	0.115	0.092	0.054	0.053	0.062	0.125	0.016	0.067	1	0	0	0
Forest	E	1.087	0.223	1.493	0.346	0.503	0.406	<0.001	0.329	<0.001	1	0	0	0
Barren	E	1.600	6.818	5.767	1.745	4.939	5.815	0.180	0.084	0.161	1	0	0	0
Shrub	E	0.902	29.152	34.075	0.418	22.542	32.286	0.016	0.098	0.146	1	0	0	0
Artificial drains	E	0.006	0.018	0.017	0.003	0.004	0.004	0.086	<0.001	<0.001	0	0	0	0
Soil permeability	E	-0.143	-0.078	-0.107	0.047	0.052	0.053	0.003	0.134	0.044	0	0	0	0
Temperature	E	-0.071	-0.084	-0.068	0.010	0.014	0.012	<0.001	<0.001	<0.001	0	0	0	0
Precipitation	E	0.014	0.015	0.010	0.001	0.003	0.002	<0.001	<0.001	<0.001	0	0	0	0
Specific catchment area	E	0.628	0.407	0.195	0.103	0.346	0.299	<0.001	0.241	0.515	0	0	0	0
Drainage density	E	0.186	0.107	0.082	0.104	0.107	0.106	0.075	0.319	0.442	0	0	0	0
Stream removal	E	17.670	10.818	23.791	2.671	6.559	6.798	<0.001	0.050	<0.001	1	0	0	0
Reservoir removal	E	1.414	1.317	2.190	0.610	0.736	0.751	0.010	0.037	0.002	1	0	0	0
Urban	NW	2.575	1.723	2.443	0.322	0.861	0.331	<0.001	0.023	<0.001	1	0	0	1
Atmospheric deposition	NW	0.694	0.000	0.749	0.144		0.161	<0.001		<0.001	1	1	0	1
Corn/soybean	NW	0.164	0.057	0.063	0.024	0.023	0.021	<0.001	0.007	0.002	1	0	0	0
Alfalfa	NW	0.082	0.059	0.048	0.058	0.057	0.041	0.076	0.152	0.125	1	0	0	1
Wheat	NW	0.137	0.000	0.000	0.060			0.011			1	1	1	0
Other crops	NW	0.071	0.048	0.047	0.030	0.026	0.017	0.010	0.033	0.003	1	0	0	1
Pasture/range	NW	0.063	0.000	0.000	0.054			0.125			1	1	1	0
Forest	NW	1.087	1.454	1.446	0.346	0.769	0.375	<0.001	0.030	<0.001	1	0	0	1
Barren	NW	1.600	67.860	77.767	1.745	34.236	22.916	0.180	0.024	<0.001	1	0	0	0
Shrub	NW	0.902	1.048	0.356	0.418	0.650	0.364	0.016	0.054	0.164	1	0	0	1
Artificial drains	NW	0.006	0.095	0.092	0.003	0.019	0.018	0.086	<0.001	<0.001	0	0	0	0
Soil permeability	NW	-0.143	-0.264	-0.132	0.047	0.098	0.044	0.003	0.007	0.003	0	0	0	1
Temperature	NW	-0.071	0.174	0.134	0.010	0.044	0.034	<0.001	<0.001	<0.001	0	0	0	0

Parameter	Region	Coefficient estimates			Standard errors[a]			p-values[a]			If coefficient subject to a physical constraint[b]	If regional fixed-effect model estimate physically constrained[c]	If hybrid model estimate physically constrained[d]	If hybrid model estimate cross-region constrained[e]
		National model	Regional fixed-effects model	Hybrid model	National model	Regional fixed-effects model	Hybrid model	National model	Regional fixed-effects model	Hybrid model				
Precipitation	NW	0.014	0.007	0.008	0.001	0.002	0.001	<0.001	0.002	<0.001	0	0	0	1
Specific catchment area	NW	0.628	-0.793	0.366	0.103	0.785	0.256	<0.001	0.313	0.153	0	0	0	1
Drainage density	NW	0.186	-0.290	0.021	0.104	0.224	0.089	0.075	0.197	0.814	0	0	0	1
Stream removal	NW	17.670	0.709	1.858	2.671	2.029	1.656	<0.001	0.364	0.131	1	0	0	0
Reservoir removal	NW	1.414	1.939	2.222	0.610	1.266	0.737	0.010	0.063	0.001	1	0	0	1
Urban	SW	2.575	3.561	3.644	0.322	0.796	0.623	<0.001	<0.001	<0.001	1	0	0	0
Atmospheric deposition	SW	0.694	0.000	0.628	0.144		0.151	<0.001		<0.001	1	1	0	1
Corn/soybean	SW	0.164	0.068	0.099	0.024	0.077	0.016	<0.001	0.189	<0.001	1	0	0	1
Alfalfa	SW	0.082	0.098	0.068	0.058	0.106	0.042	0.076	0.177	0.055	1	0	0	1
Wheat	SW	0.137	0.256	0.266	0.060	0.103	0.086	0.011	0.007	0.001	1	0	0	0
Other crops	SW	0.071	0.064	0.050	0.030	0.046	0.017	0.010	0.085	0.002	1	0	0	1
Pasture/range	SW	0.063	0.024	0.000	0.054	0.070		0.125	0.367		1	0	1	0
Forest	SW	1.087	1.717	1.255	0.346	0.591	0.373	<0.001	0.002	<0.001	1	0	0	1
Barren	SW	1.600	5.521	2.393	1.745	3.854	2.380	0.180	0.076	0.158	1	0	0	0
Shrub	SW	0.902	0.487	0.666	0.418	0.530	0.366	0.016	0.179	0.035	1	0	0	1
Artificial drains	SW	0.006	-0.004	-0.005	0.003	0.008	0.004	0.086	0.652	0.251	0	0	0	0
Soil permeability	SW	-0.143	-0.628	-0.611	0.047	0.165	0.118	0.003	<0.001	<0.001	0	0	0	0
Temperature	SW	-0.071	-0.032	-0.053	0.010	0.026	0.009	<0.001	0.233	<0.001	0	0	0	1
Precipitation	SW	0.014	0.012	0.012	0.001	0.004	0.001	<0.001	0.006	<0.001	0	0	0	1
Specific catchment area	SW	0.628	0.812	0.419	0.103	0.227	0.255	<0.001	<0.001	0.101	0	0	0	1
Drainage density	SW	0.186	-0.071	-0.068	0.104	0.264	0.088	0.075	0.787	0.441	0	0	0	1
Stream removal	SW	17.670	22.526	24.583	2.671	5.799	3.823	<0.001	<0.001	<0.001	1	0	0	0
Reservoir removal	SW	1.414	4.394	2.847	0.610	2.135	0.733	0.010	0.020	<0.001	1	0	0	1

[a] Missing values indicate the corresponding coefficient has a binding physical constraint.
[b] All source and aquatic removal rate coefficients are subject to the physical constraint that the coefficient be non-negative.
[c] Value is 1 if the regional fixed-effects model coefficient estimate is equal to the physical constraint of zero.
[d] Value is 1 if the hybrid model coefficient estimate is equal to the physical constraint of zero.
[e] Value is 1 if the hybrid model coefficient is constrained to equal a weighted average of the hybrid model coefficients for the complementary regions.

TABLE S2. Coefficient estimates and associated statistics for the total phosphorus (TP) models. See the above discussion for a description of each field presented in the table. Region abbreviations are E for East, NW for Northwest, and SW for Southwest (see Figure 1).

Parameter	Region	Coefficient estimates			Standard errors[a]			p-values[a]			If coefficient subject to a physical constraint[b]	If regional fixed-effect model estimate physically constrained[c]	If hybrid model estimate physically constrained[d]	If hybrid model estimate cross-region constrained[e]
		National model	Regional fixed-effects model	Hybrid model	National model	Regional fixed-effects model	Hybrid model	National model	Regional fixed-effects model	Hybrid model				
Urban	E	0.255	0.185	0.299	0.050	0.039	0.054	<0.001	<0.001	<0.001	1	0	0	0
Corn/soybean	E	0.023	0.033	0.029	0.012	0.018	0.013	0.029	0.033	0.015	1	0	0	0
Alfalfa	E	0.124	0.029	0.127	0.080	0.088	0.079	0.060	0.371	0.054	1	0	0	0
Other crops	E	0.026	0.034	0.040	0.016	0.025	0.018	0.053	0.081	0.013	1	0	0	0
Pasture/range	E	0.138	0.196	0.113	0.028	0.067	0.028	<0.001	0.002	<0.001	1	0	0	0
Forest	E	0.167	0.237	0.161	0.035	0.045	0.034	<0.001	<0.001	<0.001	1	0	0	0
Barren	E	1.353	0.404	1.075	0.757	0.512	0.626	0.037	0.216	0.043	1	0	0	0
Shrub	E	0.227	5.690	5.418	0.084	3.887	4.550	0.004	0.072	0.117	1	0	0	0
Artificial drains	E	0.027	0.017	0.026	0.008	0.010	0.007	<0.001	0.069	<0.001	0	0	0	0
Soil permeability	E	-0.404	-0.315	-0.397	0.074	0.082	0.073	<0.001	<0.001	<0.001	0	0	0	0
Slope	E	0.099	-0.033	0.111	0.059	0.079	0.066	0.092	0.682	0.090	0	0	0	0
Precipitation	E	0.007	0.000	0.008	0.002	0.003	0.002	<0.001	0.967	<0.001	0	0	0	0
Specific catchment area	E	0.623	0.900	0.461	0.285	0.143	0.345	0.030	<0.001	0.182	0	0	0	0
Stream removal	E	12.189	31.885	22.689	3.219	14.119	6.265	<0.001	0.012	<0.001	1	0	0	0
Reservoir removal	E	33.965	18.571	33.444	5.996	6.353	5.509	<0.001	0.002	<0.001	1	0	0	0
Urban	NW	0.255	0.284	0.294	0.050	0.217	0.053	<0.001	0.096	<0.001	1	0	0	1
Corn/soybean	NW	0.023	0.025	0.024	0.012	0.039	0.013	0.029	0.260	0.032	1	0	0	1
Alfalfa	NW	0.124	0.065	0.144	0.080	0.213	0.078	0.060	0.381	0.032	1	0	0	1
Other crops	NW	0.026	0.005	0.033	0.016	0.026	0.017	0.053	0.431	0.026	1	0	0	1
Pasture/range	NW	0.138	0.072	0.090	0.028	0.034	0.020	<0.001	0.019	<0.001	1	0	0	1
Forest	NW	0.167	0.057	0.155	0.035	0.049	0.034	<0.001	0.123	<0.001	1	0	0	1
Barren	NW	1.353	2.282	0.973	0.757	1.652	0.609	0.037	0.084	0.055	1	0	0	1
Shrub	NW	0.227	0.273	0.303	0.084	0.154	0.094	0.004	0.039	<0.001	1	0	0	1
Artificial drains	NW	0.027	0.080	0.026	0.008	0.075	0.007	<0.001	0.291	<0.001	0	0	0	1
Soil permeability	NW	-0.404	-0.385	-0.382	0.074	0.155	0.071	<0.001	0.013	<0.001	0	0	0	1
Slope	NW	0.099	-0.074	0.082	0.059	0.254	0.059	0.092	0.772	0.164	0	0	0	1
Precipitation	NW	0.007	0.008	0.009	0.002	0.003	0.002	<0.001	0.002	<0.001	0	0	0	1
Specific catchment area	NW	0.623	-1.528	0.477	0.285	1.009	0.346	0.030	0.131	0.169	0	0	0	0
Stream removal	NW	12.189	0.000	0.000	3.219			<0.001			1	1	1	0
Reservoir removal	NW	33.965	34.102	32.994	5.996	9.657	5.484	<0.001	<0.001	<0.001	1	0	0	1
Urban	SW	0.255	0.667	0.294	0.050	0.199	0.053	<0.001	<0.001	<0.001	1	0	0	1

Parameter	Region	Coefficient estimates			Standard errors[a]			p-values[a]			If coefficient subject to a physical constraint[b]	If regional fixed-effect model estimate physically constrained[c]	If hybrid model estimate physically constrained[d]	If hybrid model estimate cross-region constrained[e]
		National model	Regional fixed-effects model	Hybrid model	National model	Regional fixed-effects model	Hybrid model	National model	Regional fixed-effects model	Hybrid model				
Corn/soybean	SW	0.023	0.093	0.024	0.012	0.097	0.013	0.029	0.171	0.033	1	0	0	1
Alfalfa	SW	0.124	0.838	0.143	0.080	0.433	0.078	0.060	0.027	0.033	1	0	0	1
Other crops	SW	0.026	0.012	0.033	0.016	0.025	0.017	0.053	0.316	0.026	1	0	0	1
Pasture/range	SW	0.138	0.019	0.090	0.028	0.022	0.020	<0.001	0.191	<0.001	1	0	0	1
Forest	SW	0.167	0.001	0.155	0.035	0.030	0.034	<0.001	0.490	<0.001	1	0	0	1
Barren	SW	1.353	11.815	0.965	0.757	7.657	0.608	0.037	0.062	0.057	1	0	0	1
Shrub	SW	0.227	0.102	0.303	0.084	0.074	0.094	0.004	0.085	<0.001	1	0	0	1
Artificial drains	SW	0.027	0.027	0.026	0.008	0.013	0.007	<0.001	0.039	<0.001	0	0	0	1
Soil permeability	SW	-0.404	-0.785	-0.382	0.074	0.219	0.071	<0.001	<0.001	<0.001	0	0	0	1
Slope	SW	0.099	0.517	0.081	0.059	0.184	0.059	0.092	0.005	0.169	0	0	0	1
Precipitation	SW	0.007	0.006	0.009	0.002	0.008	0.002	<0.001	0.443	<0.001	0	0	0	1
Specific catchment area	SW	0.623	-1.935	0.447	0.285	1.371	0.344	0.030	0.159	0.194	0	0	0	1
Stream removal	SW	12.189	13.318	16.599	3.219	5.215	3.309	<0.001	0.006	<0.001	1	0	0	1
Reservoir removal	SW	33.965	38.084	33.002	5.996	10.754	5.484	<0.001	<0.001	<0.001	1	0	0	1

[a] Missing values indicate the corresponding coefficient has a binding physical constraint.

[b] All source and aquatic removal rate coefficients are subject to the physical constraint that the coefficient be non-negative.

[c] Value is 1 if the regional fixed-effects model coefficient estimate is equal to the physical constraint of zero.

[d] Value is 1 if the hybrid model coefficient estimate is equal to the physical constraint of zero.

[e] Value is 1 if the hybrid model coefficient is constrained to equal a weighted average of the hybrid model coefficients for the complementary regions.

Literature Cited

Alexander, R. B., R. A. Smith, G. E. Schwarz, E. W. Boyer, J. V. Nolan, and J. W. Brakebill, 2008, Differences in phosphorus and nitrogen delivery to the Gulf of Mexico from the Mississippi River Basin, *Environ. Sci. Technol.*, 42, 3, 822-830. DOI:10.1021/es0716103.

Amemiya, T., 1985, *Advanced Econometrics*, Harvard University Press, Cambridge, MA, 521 p.

Gallant, A. R., 1987, *Nonlinear statistical models,* Wiley, New York, 610 p.

Murphy, K. M. and R. H. Topel, 1985, Estimation and inference in two-step econometric models, *Journal of Business and Economic Statistics*, v. 3, n. 4, p. 88-97.

Schwarz, G. E., A. B. Hoos, R. B. Alexander, and R. A. Smith, 2006, The SPARROW surface water-quality model: Theory, application, and user documentation, U.S. Geological Survey Techniques and Methods Report, Book 6, Chapter B3.
